# Phytosterols and Novel Triterpenes Recovered from Industrial Fermentation Coproducts Exert In Vitro Anti-Inflammatory Activity in Macrophages

**DOI:** 10.3390/ph14060583

**Published:** 2021-06-18

**Authors:** Francisca S. Teixeira, Susana S. M. P. Vidigal, Lígia L. Pimentel, Paula T. Costa, Diana Tavares-Valente, João Azevedo-Silva, Manuela E. Pintado, João C. Fernandes, Luís M. Rodríguez-Alcalá

**Affiliations:** CBQF—Centro de Biotecnologia e Química Fina—Laboratório Associado, Universidade Católica Portuguesa, Escola Superior de Biotecnologia, Rua Diogo Botelho 1327, 4169-005 Porto, Portugal; fsteixeira@porto.ucp.pt (F.S.T.); svidigal@porto.ucp.pt (S.S.M.P.V.); lpimentel@porto.ucp.pt (L.L.P.); ptcosta@porto.ucp.pt (P.T.C.); dvalente@porto.ucp.pt (D.T.-V.); jasilva@porto.ucp.pt (J.A.-S.); mpintado@porto.ucp.pt (M.E.P.); jcfernandes@porto.ucp.pt (J.C.F.)

**Keywords:** biowaste, triterpenes, 1-octacosanol, phytosterols, stability test, molecular distillation, macrophages, inflammation, IL-6

## Abstract

The unstoppable growth of human population that occurs in parallel with all manufacturing activities leads to a relentless increase in the demand for resources, cultivation land, and energy. In response, currently, there is significant interest in developing strategies to optimize any available resources and their biowaste. While solutions initially focused on recovering biomolecules with applications in food, energy, or materials, the feasibility of synthetic biology in this field has been demonstrated in recent years. For instance, it is possible to genetically modify *Saccharomyces cerevisiae* to produce terpenes for commercial applications (i.e., against malaria or as biodiesel). But the production process, similar to any industrial activity, generates biowastes containing promising biomolecules (from fermentation) that if recovered may have applications in different areas. To test this hypothesis, in the present study, the lipid composition of by-products from the industrial production of β-farnesene by genetically modified *Saccharomyces cerevisiae* are studied to identify potentially bioactive compounds, their recovery, and finally, their stability and in vitro bioactivity. The assayed biowaste showed the presence of triterpenes, phytosterols, and 1-octacosanol which were recovered through molecular distillation into a single fraction. During the assayed stability test, compositional modifications were observed, mainly for the phytosterols and 1-octacosanol, probably due to oxidative reactions. However, such changes did not affect the in vitro bioactivity in macrophages, where it was found that the obtained fraction decreased the production of TNF-α and IL-6 in lipopolysaccharide (LPS)-induced inflammation.

## 1. Introduction

The technological advances that humankind has achieved since the first industrial revolution (i.e., 1760) to nowadays, have led to population growth, resource utilization, and environmental alterations more rapidly than recorded in previous ages. Thus, according to recent calculations, it is expected that, by 2030, the human population of the Earth could be 8.5 billion, and 9.7 billion by 2050 [1], increasing the demand for both energy and natural resources [2]. Indeed, there is significant concern regarding the future availability of fertile cropland, pure water, and energy [3,4], aggravated by climate change that disrupts society, the environment, and economics [5] in a scenario where humankind may reach a critical point in its development [6].

The need to bring sustainability policies to social, economic, and environmental features has resulted in the apparition of circular, green, and bioeconomy concepts [7]. Specifically, bioeconomy focuses on the reutilization of biological wastes from land and sea to produce food, energy, and various materials [2]. For example, processing of fruits, vegetables, dairy, bakery, and meat products, generates around 1.3 billion tons of scrap materials. In fact, by 2025, the urban quantum of wastes is estimated to be 416 million tons, whereas, in 2005, it was 278 million tons [8].

In addition to improving our efficiency of food production and distribution, recovery and reutilization are emerging as potential solutions. Several biomolecules (i.e., antioxidants, pectin, fiber, phenolics, and lipids) can still be found in such wastes, and therefore applied in animal feed [9], biomaterials [10], and sunscreen filters [11].

A paradigm in green economy is the utilization of biofuels to replace fossil oils. Thus, for bioethanol as octane additive [12], the so-called first-generation is produced during the fermentation of sugar from sugarcane or other starch-containing raw materials such as molasses [13,14]. However, this activity has recently been merged with the principles of bioeconomy, since, in second-generation bioethanol, hemicellulose and cellulose from lignocellulosic biomass, biowastes of sugarcane such as bagasse and straw, are hydrolysed to sugars and also fermented into ethanol [15]. Finally, third-generation biofuels focus on marine algae due to its high carbohydrate content [16].

This development has been motivated by the fact that production from biomass feedstock is preferable, since there is no need for extra land or competition with food production such as is expected to happen with sugarcane if the demand of this crop continues to increase according to predictions [17]. Moreover, for biodiesel elaboration, since it is composed of long chain fatty acid esters from vegetable oils and animal fats, the utilization of non-edible plant oils [18] or tallow waste [19] has also been assayed with promising results.

In a more straightforward approach to obtain valuable molecules, a solution has been proposed elsewhere based on synthetic biology where microorganisms are transformed with the appropriate set of genes [20,21]. Specifically, by genetic engineering, it has already been possible to produce terpenes and terpenoids in *Saccharomyces cerevisiae* with applications in pharmaceuticals and biofuels [22]. Artemisinin (i.e., an antimalarial drug) [23] and β-farnesene (i.e., biodiesel) [24] are examples of industrial-scale molecules produced by genetically modified *Saccharomyces cerevisiae*.

This is certainly an emerging field where the principles of bioeconomy can be applied to synthetic-biology-based processes. In fact, in the lipidome of wild type *Saccharomyces cerevisiae,* the presence of up to 160 different molecular species has been reported, where ergosterol is one of the most relevant compounds [25]. Furthermore, this sterol was able to ameliorate inflammation in diabetic nephropathy mice [26] as well as in microglia cells [27]. Moreover, it must be noted that, as previously mentioned, in general, the fermentative processes use sugar/molasses as carbon and energy sources. The producing plant (i.e., sugarcane) has been described as containing phytochemicals [28,29], also associated with anti-inflammatory properties in macrophages and mice [30]. Thus, it can be hypothesized that some of those potentially bioactive molecules could be found and recovered in the fermentation biowastes.

In the current COVID-19 pandemic scenario, the search for any available sources of bioactive lipids and mainly those with anti-inflammatory properties would be of special interest [31].

Accordingly, in this study, we aim at screening for the presence of potentially bioactive lipids in biowastes from the fermentative production of β-farnesene, and then to devise a strategy for their isolation and further characterization of both in vitro bioactivity and stability. The obtained information should allow us to unravel the suitability of industrial fermentation by-products as potential sources of lipids to be used as bioactive ingredients.

## 2. Results and Discussion

### 2.1. Composition and Presence of Potentially Bioactive Lipids in β-Farnesene Distillation Residue (FDR) Samples

During the lipid profiling of β-farnesene distillation residue (FDR), compounds have been observed by elution in the chromatographic region of glycolipids using the HPLC-ELSD technique (data not shown) with a concentration of 46.51/100 g lipids (Table 1). Such compounds are common in plant samples [32] and their presence could be related to the utilization of sugarcane syrups during the fermentative processes to produce β-farnesene, but in lower concentrations than those detected. For the purpose of confirming the presence of glycolipids, an FDR methanol fraction was analyzed by shotgun mass spectrometry in a QTOF instrument (Supplementary Materials Figure S7). The fragmentation pattern detected by the mass spectrometer revealed several ions (i.e., *m/z*) from 200 to 1400 *m/z*, and the distribution of intensities resembled that of a Gaussian distribution. Further LC-QTOF analyses showed only one eluting peak, confirming the Gaussian distribution of ions *(*Figure S8). This agrees with the presence of oligomers, suggesting that this compound can be a polymer [33]. In fact, since FDR is a by-product of the distillation of β-farnesene (PR 15), this polymer may arise during the process. Nevertheless, elsewhere, assays have successfully demonstrated the utilization of β-farnesene to produced biopolymers through anionic or cationic pathways [34] and involving redox reactions [35].

Moreover, the HPLC-ELSD analyses, in addition to the presence of polymers as mentioned above, showed that hydrocarbons were one of the major lipid compounds in an FDR sample (43.29 g/100 g lipids; Table 1). Further analysis performed by GC-MS (Table 2) confirmed that the principal lipid family consisted of terpenes, and this group was integrated by β-farnesene (PR 15, 133 g/kg, as the main compound), farnesol (PR 15:2c2t16 and PR 15:2t2t16), a sesterterpene (PR 25), and triterpenes. Regarding these latter compounds, five different triterpenes were detected in both FDR and feed samples. For their annotation, they were grouped according to their chromatographic elution (i.e., by retention time) and labeled from I to VIII (Supplementary Materials Figure S1). Afterwards, the mass spectra of the matching peaks in each sample were compared. It was found that the fragmentation pattern of peaks with the same retention time was similar, therefore, suggesting that they were the same compound. In all cases, the base peak was 69 *m/z*, while the other secondary ions were 41, 55, 81, 95, 105, 120, 133, 145, and 160 *m/z* Figures S2–S5. The highest *m/z* detected was 408 *m/z*, which may point out to be the molecular ion. Thus, compounds I to VIII seem to belong to the same family but the differences in retention time highlight that they have some structural differences.

In FDR samples, since a polymer that may arise from the polymerization of β-farnesene (PR 15) was detected, the origin of triterpenes I–VIII may also be the dimerization of this compound. When comparing the above-mentioned spectra with the one obtained after GC-MS analysis of a standard of squalene (Figure S6), the same base ion peak can be observed as well as similar secondary ions. The molecular weight of squalene is 410.71 g/mol and the possible molecular ion in compounds I–VIII had a *m/z* of 408, suggesting that, although compounds I–VIII are triterpenes, their structure is slightly different from that of squalene.

Interestingly, Fischer et al. [36] published a patent describing procedures and conditions for obtaining farnesene dimers: four dimers with molecular formula C_30_H_60_ and molecular weight of 420.84 g/mol and another four dimers with the molecular formula C_30_H_48_ (408.71 g/mol), which agrees with the characteristics of the PR 30 detected in an FDR sample.

The presence of PR 30 suggests a promising potential of FDR, since other similar triterpenes such as squalene have exhibited high biocompatibility [37] and several publications have reported that PR 30 can act as a drug carrier [38,39], allowing the transport of bioactive compounds (i.e., through the blood brain barrier [40]).

Furthermore, other bioactive lipids have also been detected by HPLC-ELSD, namely fatty alcohols (2.00 g/100 g lipids) and phytosterols (0.78 g/100 g lipids). The GC-MS analysis revealed that these lipid groups were mainly composed of 1-octacosanol (FOH 28:0, 1.45 g/kg) in the case of fatty alcohols (Table 2) and ergosterol (ST 28:3;O, 3.19 g/kg), campesterol (ST 28:1;O, 0.72 g/kg), stigmasterol (ST 29:2;O, 0.92 g/kg), and β-sitosterol (ST 29:1;O, 1.29 g/kg) for phytosterols.

Different studies have concluded that FOH 28:0 may exert bioactive properties against obesity and related metabolic disorders. When mice were exposed to a high fat diet and received 60 mg FOH 28:0/kg/day, a reduction in body weight was observed, along with an improvement in insulin resistance and hepatic lipid content [41]. Studies [42] conducted in humans have also found positive effects of FOH 28:0 supplementation on taekwondo athletes (40 mg/day for 6 days) subjected to rapid weight loss through caloric restriction, with high intensity exercise training reporting higher levels of high-density lipoprotein (HDL) with a decrease in low density lipoprotein (LDL) and TG. In addition, in mice exposed to a cage change strategy to induce mild stress and sleep disturbance, doses of 100–200 mg of FOH 28:0/kg clearly restored stress-affected sleep [43].

Phytosterols are widely recognized to be bioactive compounds that exert positive effects on human health. Thus, EU regulations regarding health claims about plant sterols accept that a human diet supplemented with 0.8 g phytosterols/day contributes to the maintenance of normal blood cholesterol levels [44].

Therefore, the performed analysis allowed us to identify the presence of compounds with potential bioactivity (FOH 28:0 and phytosterols), while PR 30 may act as a carrier and/or coadjuvant. Accordingly, the following experiments focused on the recovery of fatty alcohols, terpenes, and phytosterols in order to predict physicochemical and bioactivity parameters over an accelerated stability test.

### 2.2. Recovery of Bioactive Compounds by Molecular Distillation

At atmospheric pressure, the boiling points of β-farnesene, farnesol, and 1-octacosanol are 260, 299, and 428 °C, respectively. Regarding phytosterols, the boiling points of ergosterol and stigmasterol are 250 and 490 °C, respectively. If the detected PR 30 has, as the collected data suggest, a structure similar to squalene, then, its boiling point may be close to that of this molecule (i.e., 458 °C). Accordingly, the compounds of interest (i.e., PR 30, 1-octacosanol, and phytosterols) may be recovered together by distillation in a single fraction. This would be a promising approach since distillation could be scaled up to an industrial size.

However, these high temperatures may result in degradation of the molecules, and therefore working at a reduced pressure is required. As described in the Methods and Materials Section, the experiment was performed at 0.5–0.8 Torr, at a starting temperature of 250 °C, which was gradually increased. Prior to the distillation, FDR was degassed in a vacuum and room temperature for 30 min. This sample was labeled as Feed.

**Figure 1:**
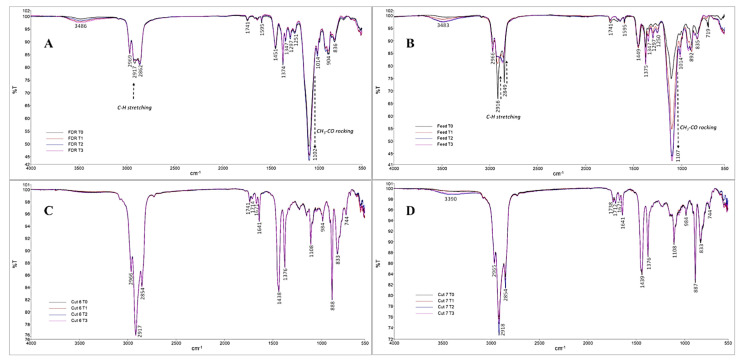
Overlay of the Fourier transform infrared spectroscopy (FTIR) spectra of FDR (**A**), Feed (**B**), C6 (**C**), and C7 (**D**) samples throughout the stability test (T0, T1, T2, and T3).

As a first approach, the FDR and Feed samples were compared by FTIR-ATR (Figure 1). Thus, it was possible to detect a band at 3486 cm^−1^ inherent to the –OH stretching vibration due to the presence of alcohols, namely farnesol. At 1741 cm^−1^, there was evidence of the presence of a –C=O stretching vibration band, which might be related to the presence of carboxylic acids, esters, ketones, or aldehydes. At 1297 cm^−1^, the –CH deformation vibration band was identified and may be related to the presence of unsaturated aliphatic chains, probably from unsaturated farnesene dimers, according to structures described elsewhere [36].

According to the gas chromatographic analyses (Table 2), it was observed that the β-farnesene content in the Feed sample was slightly lower than in that the FDR sample (133.48 g/kg FDR vs. 116.09 g/kg Feed, *p* > 0.05). However, the contents of FOH 28:0 were significantly affected after the vacuum degassing step, i.e., 1.45 g/kg FDR vs. 0.25 g/kg Feed. This difference was also observed in the data obtained by HPLC-ELSD, as fatty alcohols levels in the Feed sample were 1.75 g/100 g and 2.00 g/100 g (*p* > 0.05) in the FDR sample.

There were no further alterations observed after the degassing step. Thus, according to the principal component analysis (PCA) (Supplementary Materials Figure S9) of the data obtained by GC-MS and HPLC-ELSD, both the FDR and Feed samples cluster on the positive side of the x-axis (PC1), which indicates similarity between these samples.

FOH 28:0 is the main component of policosanol, a mixture of long chain fatty alcohols that can be found in the waxes of sugarcane [45], rice bran [46], wheat and flax straw [47], and other plant materials. Studies conducted on rice bran oil stored for 17 days at 40 °C, have found that only triglycerides were affected by oxidation due to the intramolecular formation of hydroperoxides, mainly in molecular species containing linoleic acid [48]. When FOH 28:0 and phytosterols were isolated from sugarcane rind and heated up to 300 °C, 21 degradation compounds from both FOH 28:0 and phytosterols were found, which were not identified [49]. These latter authors reported that the oxidation onset temperature for FOH 28:0 was 245.74 °C, according to differential scanning calorimetry tests.

It has been proposed that the oxidation of fatty alcohols occurs through a first step where the corresponding aldehyde is formed, to afterwards being acetalized with the fatty alcohol yielding the hemiacetal, whose oxidation directly produces a wax ester, without the intervention of a fatty acid [50]. However, such reactions at room temperature need a catalyst [51]. According to the obtained results in this study, in the FDR samples, the wax ester content was 0.22 g/100 g, while for the Feed sample it was 0.11 g/100 g (*p* < 0.05). However, the contents of triglycerides (4.21 g/100 g vs. 4.34 g/100 g) and free fatty acids (0.33 g/100 g vs. 0.30 g/100 g) were not altered. The fact that the phytosterol content in FDR was 6.11 g/kg and 6.52 g/kg in Feed (*p* > 0.05) seems to indicate that oxidation reactions were not taking place. Moreover, in the assayed conditions, aldehydes were not detected, or any other degradation compounds related to fatty alcohols. Nevertheless, it could be hypothesized that, although octacosanal has a boiling point of 410 °C, under the vacuum conditions carried out during the degassing step, it could evaporate. Thus, while FOH 28:0 disappeared, its oxidation intermediates would not be detected.

Alternatively, as farnesene and farnesol contents were slightly lower in the Feed samples than in the FDR samples, another explanation could be that the conditions assayed during the degassing step allowed the evaporation of some compounds, including FOH 28:0. Thus, this second explanation seems to be more likely, since according to previous information, FOH 28:0 is highly resistant to degradation, and this is only observed under harsh conditions.

After the degassing step, the obtained sample (i.e., Feed) was assayed in the spinning band. Under the tested conditions, a total of seven fractions (i.e., cuts) were collected but only those corresponding to C6 and C7 did not contain farnesene or farnesol isomers (Table 3). They were also the only distillates showing the presence of the target bioactive lipids: PR 30 (753.64 g/kg in C6 and 634.20 g/kg in C7, *p* < 0.05), FOH C28:0 (1.61 g/kg in C6 and 3.88 g/kg in C7, *p* < 0.05), and ST (11.40 g/kg in C6 and 30.84 g/kg in C7, *p* < 0.05). Thus, C6 showed higher contents of PR 30 than C7, being the main compounds, triterpene IV (438 g/kg in C6 and 352 g/kg in C7, *p* < 0.05), and triterpene VIII (242 g/kg in C6 vs. 230 g/kg in C7). These two triterpene compounds (IV and VIII) were also the most relevant of this group in terms of concentration in the FDR sample. However, triterpenes II, V, and VII were only detected after distillation in C6 and C7 samples. They were probably present in the FDR sample but in concentrations below the LOD of the technique.

Regarding the ST moiety, the compound profile was the same in C6 and C7 and in agreement with that previously mentioned for FDR (i.e., ergosterol, campesterol, stigmasterol, and β-sitosterol). Regarding the profile of other lipid classes and other compounds detected by HPLC-ELSD (Table 4), the polymer was eliminated during the distillation process as well as monoglycerides.

### 2.3. Stability of the Distillates Enriched in Bioactive Lipids

The distillation procedure assayed on the Feed samples resulted into two fractions enriched in PR 30, and the potentially bioactive lipids FOH 28:0 and ST (i.e., C6 and C7). At this point, an accelerated stability study was conducted to observe possible alterations that these materials might undergo, with special interest in the mentioned bioactive lipids. The obtained data would help to understand potential applications of the distillates as functional ingredients.

According to the results obtained by FTIR-ATR (Figure 1), for the FDR and Feed samples, it was possible to verify a decrease in the intensity of the band at 2969–2849 cm^−1^ (–CH stretching, corresponding to aliphatic chains), accompanied by an increase in the intensity of the bands at 1108–1102 and 1014 cm^−1^ (–CH_3_-CO rocking, corresponding to the ketone groups) from T0 to T3. These variations suggest that, during the stability test, the oxidation of farnesene to a ketone was occurring [52].

Moreover, for the C6 and C7 samples, the obtained spectra at T0 showed a vibration band at 1714–712 cm^−1^ (–OH bending) due to the presence of 1-octacosanol and phytosterols. The observations of two vibrational bands at 1671 cm^−1^ and 1641 cm^−1^ (–C=C–stretching vibration) and another band at 888 cm^−1^ (–CH_2_ out of plane deformation) indicate the presence of aliphatic unsaturated chains, probably associated with terpenes. Furthermore, the overlapping of T0, T1, T2, and T3 FTIR-ATR spectra for both samples (C6 and C7) showed that there were no changes in their physicochemical profiles throughout time.

Regarding the lipid compositions of the C6 and C7 samples, during the accelerated study, it was found a decrease in the concentration of phytosterols (Table 4. For the C6 sample, the phytosterols content at T0 was 1.32 g/100 g and at T3 it was 1.02 g/100 g (*p* < 0.05). For the C7 sample, the initial concentration (i.e., T0) was 3.40 g phytosterols/100 g and, at the end of the assayed sampling time, it was 2.89 g phytosterols/100 g (*p* < 0.05).

The decrease in phytosterol content mentioned above did not follow the same pattern for both distillates. For C6, the reduction was only significant after T1 (1.13 g phytosterols/100 g in T2 samples), while for C7, the alteration was observed at the first sampling point (3.30 g phytosterols/100 g in T1 samples). The reduction in phytosterol content also occurred in the FDR sample (0.78 g/100 g in T0 vs. 0.36 g/100 g in T3, *p* < 0.05) and the Feed sample (0.73 g/100 g in T0 vs. 0.37 g/100 g in T3, *p* < 0.05) (Table 4).

Due to the antioxidant properties of these compounds, such loss may indicate that oxidation reactions are taking place [49]. Phytosterols can be oxidized through auto- or photo-oxidation resulting in phytosterols oxidation products (POPs) [53]. On the one hand, these POPs can be nonpolar when derived by oxidation, dehydrogenation, or dehydration and, on the other hand, polar when derived by epoxidation, hydration, or interaction with oxygen or reactive free radicals. Possibly, for all these reactions, the target could be the steroid ring. Furthermore, Lengyel et al. [54] suggested that the position of the double bonds drastically affected the stability of the molecule since Δ7 sterols were more susceptible to oxidation attack than Δ5.

For all the assayed samples, the phytosterol fraction was composed of ergosterol, campesterol, stigmasterol, and β-sitosterol. In both the FDR and Feed samples, only ergosterol decreased at the end of the assayed time. In both distillates, all phytosterols were affected, but ergosterol showed the highest reduction rates (i.e., 57.48% in C6 and 63.08% in C7). Interestingly, these compounds have double bonds at positions 5 and 7 and these results seems to agree with the hypothesis proposed by Lengyel et al.

Some other lipids detected in the assayed samples are known to be prone to undergo oxidation or to be involved in lipid degradation as free fatty acids. Lipid peroxidation reactions have such compounds as substrates: during initiation, fatty acids are converted into lipid alkyl radicals that react with ^3^O_2_ to yield lipid peroxy radical (i.e., propagation phase) to finally subtract a hydrogen to produce hydroperoxides [55]. These latter compounds can further be transformed into aldehydes, ketones, acids, esters, alcohols, and short-chain hydrocarbons.

The variations of free fatty acids from T0 to T1 were as follows: 0.46 g/100 g vs. 0.60 g/100 g in the C6 sample and 0.82 g/100 g vs. 1.11g/100 g in the C7 sample (*p* < 0.05). Such increments were also detected in the FDR sample (0.33 g/100 g at T0 vs. 0.48 g/100 g at T3, *p* < 0.05) but not in the Feed sample. An explanation for this could be the hydrolysis of lipids presenting esterified fatty acids in their structure as triglycerides, diglycerides, or wax esters. However, in the case of the triglycerides, those changes only started after T1. It must be noted that as a result of oxidation, the fatty acid backbone of triglycerides can transform into hydroperoxides [48] and, afterwards, yield oxidized monomers, dimers, and oligomers in concentrations inversely correlated to those of free fatty acids [56].

Other study results have shown that the matrix plays an important role in the oxidative stability of lipids. On the one hand, in commercial infant formulas containing vegetable oils, changes in oxidation markers (i.e., oxidized triglyceride monomers and free fatty acids) were not detected over 4 years [57] or by accelerated storage [58]. On the other hand, studies conducted on crude palm oil stored for 12 months at 26–32 °C, 20–25 °C, and 4–8 °C reported that acid value and peroxide value increased in all the assayed conditions due to microbiological growth and moisture, although those parameters were not determined [59]. Furthermore, when roasted coffee was monitored over 6 months, in the presence of air or nitrogen at 30 °C or 5 °C, after the first month of storage, an increment in the concentration of free fatty acids was found that was consistent with triglycerides hydrolysis, and afterwards, a decrease during the assayed time as a result of oxidation [60]. This also agrees with studies conducted in rice bran (i.e., natural source of 1-octacosanol), where crude samples at 20–25 °C for 24 weeks showed free fatty acid increments above those observed when rice was processed using heating treatments [61]. Those authors hypothesized that heating inactivates residual enzymes (i.e., lipases).

Other glycerolipids that were detected in the assayed samples of this study were diglycerides. On the one hand, their contents remained unchanged in the C7 samples, although a slight increase was observed after T1 (*p* > 0.05) which seemed to be consistent with the hydrolysis of triglycerides. On the other hand, for C6 samples, the levels decreased significantly from T0 to T1 (3.01 g/100 g vs. 2.42 g/100 g) and remained constant during the two following sampling times, to decrease at T3 to 2.01 g/100 g (*p* < 0.05). The FDR and Feed samples showed reductions in diglycerides after T0.

Wax esters are lipids composed of a fatty acid esterified to a fatty alcohol. In the FDR and Feed samples, these lipids significantly decreased after T2. Interestingly, in both the C6 and C7 samples, the concentration of wax esters significantly increased from T0 to T1, decreased afterwards, and increased again in T3. As previously explained in the above sections, wax esters can be created through the oxidative degradation of 1-octacosanol [50]. Indeed, the concentration of 1-octacosanol changed in the C6 sample from 1.61 g/kg at T0 to 1.49 g/kg at T1 while it was not detected in the following sampling points. For the C7 sample, the concentration decreased from 3.88 g/kg at T0 to 3.43 g/kg at T1 and was also not detected at T2 or T3.

Therefore, the obtained results suggest that during the assayed accelerated storage test, for both C6 and C7 samples, oxidation reactions take place resulting in a complete loss of 1-octacosanol, which may be converted into wax esters, reduction in the phytosterol content, and a probable hydrolysis of triglycerides.

### 2.4. Bioactivity Studies of C6 and C7 Samples

**Figure 2:**
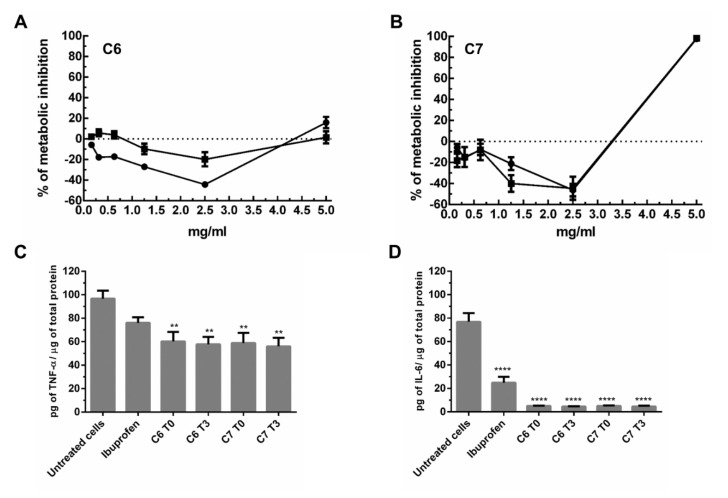
Effect of the distilled samples on macrophages. (**A**,**B**) Cytotoxicity evaluation of C6 and C7 by measuring the metabolic inhibition (PrestoBlue assay). Dots correspond to T0 samples and squares correspond to T3 samples (**C**,**D**) evaluation of TNF-α and IL-6 levels, respectively, in macrophages under an inflammatory stimulus (LPS). Values are expressed as pg of cytokine normalized to the total protein content. Ibuprofen at 1 mM was used as an anti-inflammatory control. One-way ANOVA was used for statistical analysis. ** *p* ≤ 0.01; **** *p* ≤ 0.0001.

The distilled samples, i.e., the C6 and C7 samples at the beginning and the end of the stability study, T0 and T3, respectively, were used to evaluate cytotoxicity on macrophages (Figure 2A,B, respectively). In both samples, no significative differences were observed between T0 and T3, which indicated that the samples did not degrade into toxic compounds with time. Sample C6 did not present cytotoxicity at any concentration tested. Although sample C6 at T0 showed lower values of metabolic inhibition than at T3, no conclusions could be made. The negative values of metabolic inhibition might indicate an increase in cellular proliferation; however, additional oriented studies should be conducted to pursue such a hypothesis. Sample C7 presented cytotoxicity at the highest concentration tested (5 mg/mL) with no differences observed between T0 and T3 (Figure 2B).

**Figure 3:**
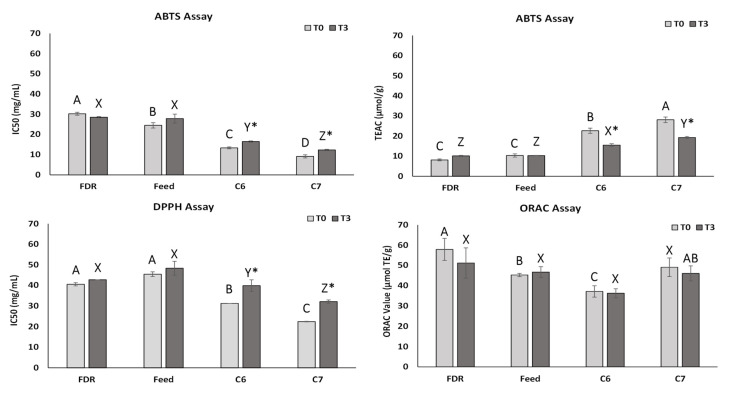
Antioxidant values determined by ABTS, DPPH, and ORAC assays for the FDR, Feed, C6, and C7 samples over the accelerated stability storage (T0 vs. T3). A,B,C letters on top of bars for significant differences (*p* < 0.05) among samples at T0. X,Y,Z for significant differences (*p* < 0.05) at T3. * significant differences (*p* < 0.05) along time for a same sample (i.e.. T0 vs. T3).

The distilled samples, C6 and C7, at T0 and T3, at 2 mg/mL, were used to evaluate cytokine production by macrophages under regular or inflammatory conditions (without and with LPS stimulus, respectively). In the Supplementary Materials the values of IL-β, IL-6, IL-8, and TNF-α normalized to total protein content are shown. For IL-β and IL-8, no differences were observed under the conditions tested, although samples C6 at T0 and T3 and sample C7 at T0 did increase the concentration of IL-8 in macrophages under no stimulus. For IL-6 and TNF-α, no differences were observed in macrophages without inflammatory stimulus. However, under LPS stimulation, all the samples tested lead to statistically significant decreases in TNF-α and IL-6 levels (C and D in Figure 3). There were no differences in anti-inflammatory activity found between T0 and T3 for both C6 and C7 distilled samples.

The influence of the lipidic extracts resulting from distillation of samples C6 and C7 were analyzed in macrophages. Contrary to the Feed sample which was highly cytotoxic to cells (i.e., 100% metabolic inhibition at any assayed concentration independent of sampling at T0 or T3), both C6 and C7 samples presented no toxicity, up to 5 and 2.5 mg/mL, respectively. Furthermore, the analysis of cytokine production in the absence and the presence of an inflammatory stimulus (LPS) revealed that both samples strongly decreased the levels of TNF-α and IL-6, which demonstrated a possible anti-inflammatory activity by these extracts. The anti-inflammatory capacity of most of the constituents identified in these extracts have already been described elsewhere [30,62,63]. Nevertheless, the reported results also show that these extracts present a high level of stability over time with respect to anti-inflammatory activity. Although the levels of fatty alcohols and sterols change with time, the capacity of these extracts to decrease the levels of TNF-α and IL-6 in an inflammation context is retained.

### 2.5. Changes in Antioxidant Activity of the Assayed Samples during the Stability Test

The obtained results regarding the antioxidant performance of samples by the DPPH, ABTS, and ORAC assays are represented in Figure 3.

The values obtained for the DPPH assay showed that the C6 and C7 samples presented higher antioxidant activity than the Feed and FDR samples at the T0 sampling time, since these last two samples had higher IC_50_ values (*p* < 0.05). The antioxidant activities of the C6 and C7 samples were slight reduced during the stability test (*p* < 0.05), which were probably related to decreases in phytosterol content in these samples from T0 to T3, therefore, suggesting that those changes are related to oxidation reactions. This behavior is reflected by an increase in the IC_50_ values (from 31.26 ± 0.12 mg/mL at T0 to 39.87 ± 2.81 mg/mL at T3 and from 22.43 ± 0.17 mg/mL at T0 to 32.12 ± 0.79 mg/mL at T3, respectively, for the C6 and C7 samples). The FDR and Feed antioxidant activities did not change significantly during the stability test.

In a similar way to the DPPH assay, the ABTS test results showed that the C6 and C7 samples presented higher antioxidant activity than the Feed and FDR samples at T0. On the one hand, the ABTS values indicated that the distillates had a slight decrease in antioxidant activity from T0 to T3, established by an increase in the IC_50_ values (from 13.35 ± 0.51 mg/mL at T0 to 16.47 ± 0.34 mg/mL at T3 and from 9.16 ± 0.78 mg/mL at T0 to 12.30 ± 0.31 mg/mL at T3, respectively, for the C6 and C7 samples). As mentioned before, these differences between T0 and T3 are probably related to a decrease in the phytosterol content in these samples. On the other hand, the FDR and Feed antioxidant activities did not vary at the different assay times.

Considering the ORAC values of all assayed samples, these did not change over time (37.15 μmol TE/g at T0 vs. 36.25 μmol TE/g at T3, 49.07 μmol TE/g vs. 46.03 μmol TE/g, 45.24 μmol TE/g vs. 46.65 μmol TE/g and 57.87 μmol TE/g vs. 51.17 μmol TE/g, respectively, for the C6, C7, Feed and FDR samples).

Therefore, after distillation, the obtained material (C6 and C7 samples) showed higher antioxidant capacity than the starting materials (FDR and Feed samples).

### 2.6. Crystallization, Melting, and Decomposition Temperature

The measured melting and crystallization temperatures for the FDR and Feed samples at T0, were 44.1 °C and 39.4 °C and 40.9 °C and 29.6 °C, respectively (Table 5). The fact that these transitions were not detected at T1, T2, and T3 sampling times, may be due to the decreasing amounts of farnesene, fatty alcohol, and wax esters, previously discussed in the above section.

Interestingly, the enthalpy values involved in both transitions (melting and crystallization) were slightly lower in the Feed sample than that of the FDR sample, i.e., melting enthalpy was 6.4 J/g FDR and 4.3 J/g Feed while for crystallization was 4.7 J/g FDR vs. 1.6 J/g Feed. These results suggest that the slight differences regarding composition found for these two samples affected their thermal characteristics.

Furthermore, the DSC analysis revealed that both samples, FDR and Feed, decomposed at 400.5 °C and 400.7 °C, respectively. On the one hand, the decomposition temperatures did not vary significantly during the stability test, i.e., 403.9 °C for the FDR sample and 399.4 °C for the Feed sample. On the other hand, the enthalpy values presented for this transition were quite variable, i.e., from 317.2 J/g to 553.9 J/g for the FDR sample and from 348.4 J/g to 409.7 J/g for the Feed sample.

Regarding the distilled samples, the DSC analyses (Table 5) showed that the C7 sample presented higher crystallization and melting temperatures than that of the C6 sample (crystallization temperatures of 28.6 vs. 37.2 °C and melting temperatures of 41.5 °C vs. 46.4 °C, for C6 vs. C7, respectively). According to the characterization obtained by GC-MS (Table 3), this was probably due to the higher concentration of 1-octacosanol and phytosterols (heavier compounds) in the C7 sample. These properties (crystallization and melting temperatures) did not show much alteration during the tested time (i.e., from T0 to T3): C6 crystallization and melting temperatures varied from 28.6 °C to 26.7 °C and from 41.5 °C to 35.4 °C, respectively, from T0 to T3; C7 crystallization and melting temperatures varied from 37.2 °C to 35.8 °C and from 46.4 °C to 44.2 °C, respectively, from T0 to T3.

Finally, regarding the decomposition temperatures of samples C6 and C7 during the stability test, the values were from 365.3 °C at T0 to 383.9 °C at T3 and from 370.9 °C at T0 to 377.4 °C at T3, for the C6 and C7 samples, respectively. Similar to what happened with the FDR and Feed samples, the enthalpy values associated with the decomposition process ranged from 172.9 J/g to 215.5 J/g for the C6 sample and from 117.9 J/g to 297.3 J/g for the C7 sample.

## 3. Materials and Methods

### 3.1. Materials and Chemicals

Farnesene distillation residue (FDR, collected in January 2020), a biowaste of the fermentative production of β-farnesene, was kindly donated by Amyris Inc., (Campinas, Brazil). Feed (FDR after pretreatment for distillation) and the distillates of interest (Cut 6 (C6) and Cut 7 (C7)) were also assayed.

For the HPLC assays, the mobile phases were prepared with the following solvents: 2-propanol (LC-MS grade ≥ 99.9%), isooctane (HPLC grade ≥ 99.8%) purchased from VWR Chemicals (Radnor, PA, USA), acetone (AcO) (HPLC grade ≥ 99.8%) purchased from Thermo Fisher Scientific (Waltham, MA, USA), ethyl acetate (EtAc) (HPLC grade ≥ 99.7%), water (for HPLC) purchased from Honeywell (Charlotte, NC, USA), acetic acid (HPLC grade ≥ 99.8%) purchased from Carlo Erba Reagents (Barcelona, Spain), and triethylamine (TEA) (grade ≥ 99.5%) purchased from Merck (Darmstadt, Germany). The samples were dissolved in dichloromethane (DCM) (HPLC grade ≥ 99.9%) from VWR Chemicals (Radnor, PA, USA).

For the GC-MS analysis, the internal analytical standard dodecane (99%) and the derivatizing reagent N,O-bis(trimethylsilyl)trifluoroacetamide with 1% trimethylchlorosilane (BSTFA) were purchased from Merk (Darmstadt, Germany).

Regarding the antioxidant assays, a phosphate buffer solution (75 mM, pH 7.4) was prepared by using the salt solution of sodium dihydrogen phosphate (NaH_2_PO_4_) anhydrous, ≥98% assay, purchased from Merk (Darmstadt, Germany).

Trolox^®^ (6-hydroxy-2,5,7,8-tetramethylcroman-2-carboxilic acid) was used as radical scavenging and antioxidant standard, obtained from Merk (Darmstadt, Germany) with ≥97% assay. Stock methanolic solution of DPPH^•^ (2,2’-diphenyl-1-picrylhydrazyl) was prepared using DPPH^•^ (95%), from Thermo Fisher Scientific (Waltham, MA, USA), and the final DPPH^•^ concentration was determined spectrophotometrically (final absorbance of 0.600 ± 0.100 at 515 nm). The ABTS diammonium salt (2,2′-azinobis(3-ethylbenzothiazoline-6-sulfonic acid) (≥98%), purchased from Merk (Darmstadt, Germany), was dissolved in ultrapure water and oxidized by aqueous solution of potassium persulfate (K_2_O_8_S_2_) ≥99.0%, purchased from Merk (Darmstadt, Germany). The mixture was protected from light and mixed for 16 h in order to obtain the radical cation ABTS^•+^.

For the ORAC measurements, the free radical initiator 2,2′-azobis-(2-methylpropionamidine) dihydrochloride (AAPH) (97%) was obtained from Merk (Darmstadt, Germany) as well as fluorescein disodium salt used as a fluorescent probe.

### 3.2. Distillation

Initially, 500 g of the FDR sample was degassed under vacuum, for 30 min, as a sample pretreatment, in order to eliminate water and possible gases, named the Feed sample. The recovery of target compounds was performed in a Mini Fractional Crude Oil Distillation system (B/R Instrument Corporation, Easton, PA, USA) under 0.5–0.8 Torr. The fractionation experiment was conducted with 480 g of Feed, placed in a round flask and attached to the system. The first fraction started to distillate at 250 °C, then, the temperature was increased to 265 °C, and the content was collected. A 10-minute equilibration step was performed and followed by a new temperature increase until the next fraction started to distillate. All fractions were collected in tared round glass flasks.

A total of 7 distillates were collected and labeled as Cut 1 to 7 (C1 to C7). Samples C6 and C7 were selected for further studies. Those samples were obtained at temperature intervals of 375–410 °C and 410–425 °C, respectively.

### 3.3. Accelerated Stability Test

In order to evaluate the stability of the mentioned samples under the influence of environmental factors such as temperature and humidity, an accelerated stability test was conducted at 50 °C and 75% of humidity conditions, according to the guidelines of the European Medicines Agency [64].

The FDR, Feed, C6, and C7 samples were weighed (50 mg) in triplicate and placed into separated vials. The vials designated as T0 were collected and stored at −80 °C for further analysis. The remaining vials were placed into a desiccator, in which the desiccant salt was replaced by a saturated aqueous solution of sodium chloride in order to maintain the humidity at 75% [65]. The desiccator was covered and kept in an oven at 50 °C, for 3 months.

After the first month, the vials identified as T1 were collected, purged with nitrogen, and stored at −80 °C. At the end of the second and third months, the same procedure was repeated for the vials identified as T2 and T3, respectively.

All samples were characterized as to their composition (HPLC-ELSD and GC-MS), physicochemical profile (FTIR-ATR), thermal properties (DSC), as well as antioxidant and anti-inflammatory activity (the latter activities were performed only for T0 and T3 sampling times).

### 3.4. HPLC-ELSD Analyses

The samples were accurately weighed and dissolved in dichloromethane at a concentration of 3 mg/mL. Then, samples were analyzed on an HPLC (model 1260 Infinity II, Agilent Technologies, Santa Clara, CA, USA) attached to an Evaporative Light Scattering Detector (ELSD, 1290 Infinity II, Agilent Technologies, Santa Clara, CA, USA) using nitrogen as nebulizing gas coupled to a Zorbax RX-SIL column (2.1 × 150 mm, 5 µm, Agilent Technologies, Santa Clara, CA, USA). The analysis conditions were assayed, as described by Abreu et al. [66], with slight changes (please see Supplementary Materials data. The compositions of the mobile phases were as follows: (A) isooctane/ethyl acetate (99.8:0.2, *v*/*v*); (B) acetone/ethyl acetate (2:1, *v*/*v*) containing 0.1% acetic acid (*v*/*v*); (C) 2-propanol/water (85:15, *v*/*v*) containing 0.013% acetic acid (*v*/*v*) and 0.031% of TEA *v*/*v*; (D) EtAc. The flow rate was set at 0.275 mL/min and an injection volume of 20 µL. The detector was set as follows: evaporator and nebulizer temperature set at 60 °C with nitrogen as nebulizing gas at 1.20 SLM flow rate. For the determination of elution order, pure standards were injected as well as available bibliography was used [67]. In all analyses performed, all the samples were injected at least in triplicate.

### 3.5. GC-MS Analyses

The samples were previously derivatized into their trimethylsilyl derivatives (TMS). Thus, in a glass vial, 5 mg of lipid extract were accurately weighted and added with 100 µL dodecane (2.5 mg/mL in DCM), 30 µL of BSTFA and DCM to a final volume of 1.3 mL. The mixture was incubated for 60 min at 30 ° C.

Derivatized samples were analyzed on a GC-QqQ model EVOQ (Bruker, Karlsruhe, Germany) mass spectrometer, with a Rxi-5Sil MS column (30 m × 250 µm × 0.25 µm nominal) at constant flow of 1 mL/min. The carrier gas used was helium and the operating conditions were as described by Attard et al. [45], with some modifications. The injector was set at 340 °C with a spilt 10, and the oven temperature started at 60 °C with a hold for 1 min, then, the temperature was increased at 5 °C/min until 340 °C and maintained for 10 min. The mass spectrometer detector was operated in electron ionization mode (EI) at 70 eV, a source temperature of 280 °C, the transfer line at 300 °C, and a quadrupole in a scan range from 33 to 1000 amu per second. The compound identification was based on the comparison of the obtained mass spectra with the information on the NIST library (v. 2.3) as well as by comparison with reference compounds.

To calculate the limits of detection (LOD) and quantification (LOQ), a calibration curve of dodecane was prepared in triplicate as TMS assaying concentrations in the range from 0.020 to 1 mg/mL. R^2^ was 0.999, LOD was 24 µg/g, and LOQ was 74 µg/g. For all the analyses, all samples were injected at least in triplicate.

### 3.6. Fourier Transform Infrared Spectroscopy with Attenuated Total Reflectance (FTIR-ATR)

The samples were analyzed on a PerkinElmer Paragon 1000 FTIR (Waltham, MA, USA) with an ATR accessory (Diamond/ZnSe). The spectra were obtained in the wavenumber range of 4000–550 cm^−1^, with a resolution of 4 cm^−1^, by accumulating 16 scans. The FTIR-ATR vibrational bands were identified based on the literature [68] and are summarized in the Supplementary Materials (Table S2).

### 3.7. Cell Culture

The human monocytic cell line THP-1 (ATCC TIB-202) was kept in culture in RPMI media (Gibco) supplemented with 10% FBS (Gibco), 1% antibiotic (Gibco), and 50 mM of beta-mercaptoethanol (Gibco) at 37 °C, with 5% CO_2_ in a humidified atmosphere. For the experiments, the THP-1 cells were seeded according to the respective plate and differentiated into macrophages by treatment with 50 nM of phorbol 12-myristate 13-acetate (PMA, Sigma, St. Louis, MO, USA, EUA) for 48 h.

### 3.8. Cytotoxicity Assays

Cytotoxicity of lipidic extracts on macrophages were evaluated using a PrestoBlue assay (Thermo Fischer), according to the manufacturer’s instructions. The THP-1 cells were seeded, at 1 × 10^4^ cells/well, in 96-well plates and differentiated into macrophages. Then, cells were exposed to the lipidic extracts at different concentrations for 24 h, in quadruplicates. Wells with media supplemented with lipidic extracts (without cells) were used to subtract a possible influence of the samples in the PrestoBlue fluorescence signal. Cells treated with 10% DMSO were used as a negative control. After incubation, PrestoBlue reagent was added to the media and incubated for 2 h. The fluorescence signal was read in a Synergy H1 microplate reader (BioTek). The results are expressed in percentage of metabolic inhibition as compared with a control (cells without treatment). At least two independent experiments were performed.

### 3.9. Immune Response

The THP-1 cells, seeded at 3 × 10^5^ cells/well in 24-well plates, were differentiated into macrophages. Cells were treated for 24 h with the lipidic extracts at 2 mg/mL in the presence or absence of lipopolysaccharides from *Escherichia coli* O111:B4 (LPS, Sigma) at 1 µg/mL to induce inflammation. For anti-inflammatory control, cells were treated with 1 mM of Ibuprofen (Mylan). Medium supernatants were collected and used to evaluate the levels of proinflammatory cytokines IL-6, IL-8, TNF-α, and IL-β by ELISA (Biolegend, San Diego, CA, USA). Cells were lysed with water and used for protein quantification via BCA method (Thermo Fischer, Waltham, MA, USA, EUA). The results were expressed in pg of cytokine/µg of total protein. At least two independent experiments were performed.

### 3.10. Antioxidant Activity

For the DPPH microplate method, the free radical scavenging activity was determined spectrophotometrically. The 2,2’-diphenyl--picrylhydrazyl radical (DPPH^•^) analysis conditions were performed in a Greiner Bio-One transparent 96-well microplate (North Carolina, USA) and assayed as described by Bobo-García et al. [69], with some modifications. Samples were prepared from a stock solution (40 mg/mL) with consequent several dilutions from 28.00 to 0.88 mg/mL in methanol. The stock solution was submitted to ultrasounds (10 min) in order to improve the dissolution, and then filtered using Macherey-Nagel 0.45 µm pore size Chromafil^®^ PET filters (Düren, Germany). The absorbance of the mixture from a stable violet at 515 nm to yellow after the addition of different quantities of sample/standard was measured. Trolox (0.0075–0.075 mg/mL) was used as antioxidant standard. A total of 25 μL of the sample, standard or methanol (negative control), was added in each well separately to 175 μL of DPPH^•^ methanolic solution (60 µM) and shaken for 5 s in the Synergy H1^TM^ microplate reader (BioTek Instruments, Inc.). After 30 min incubation period at 23 °C kept in the dark, the absorbance was measured at 515 nm on a Synergy^TM^ microplate reader, and the experiment was carried out in triplicate.

The inhibition capacity expressed as *IC*_50_ values indicate the concentration of the antiradical compound necessary to decrease 50% of the initial DPPH^•^ absorbance and was calculated using the linear regression from the concentration of sample versus percentage of inhibition [70]. To calculate the scavenging capability of DPPH radical, both DPPH^•^ percentage (%) of inhibition and *IC*_50_ were calculated by the following equations, respectively:

(1) $$\%\;Inhibition=\frac{A_{negative\;control\;}-A_{sample\;}}{A_{negative\;control\;}}\times 100$$

(2) $$IC_{50}\;(\mathrm{mg}/\mathrm{mL})\;=\frac{50-b}{m}$$

The ABTS assay was performed in a Greiner Bio-One transparent 96-well microplate (Kremsmünster, Austria), based on the inhibition by antioxidants of the absorbance of the radical cation 2,2-azinobis-(3-ethylbenzothiazoline-6-sulphonate) (ABTS^•+^), which has a characteristic wavelength absorption spectrum at 734 nm [71].

The samples used in this method were prepared the same as for the DPPH^•^ method mentioned above and the analysis conditions were assayed as described by Benteldjoune et al. [72], with some modifications. Briefly, the 20 µL of sample, standard or methanol (negative control), was added in each well separately and allowed to react with 180 µL of ABTS^•+^ solution (prepared in methanol to an absorbance of 0.700 ± 0.02 at 734 nm) for 5 min in the dark and the absorbance was immediately recorded at 734 nm by a Synergy H1^TM^ microplate reader (BioTek Instruments, Inc.) and the experiment was carried out in triplicate. Trolox diluted in phosphate buffer was used as the antioxidant standard (25–175 µM). The Trolox equivalent antioxidant capacity (*TEAC*) was calculated using the following equation:

(3) $$TEAC\;\left(µ\mathrm{mol}/g\right)=\frac{\left(\frac{\left(IC{\%}_{Sample}-b\right)}{a}\times 10^{6}\right)}{C_{Sample\;\;}\times M_{Trolox}}$$

To perform the ORAC microplate method, samples were prepared from a stock solution (2.5 mg/mL) with consequent several dilutions from 2.00 to 0.06 mg/mL in a phosphate buffer (PBS) (75 mM, pH 7.4). The stock solution was submitted to ultrasounds (10 min) in order to improve the dissolution and then filtered using Macherey-Nagel 0.45 µm pore size Chromafil^®^ PET filters (Düren, Germany). The analysis conditions were assayed as described by Dávalos et al. [73] with some modifications. In a Thermo Fisher 96-well black microplate (Waltham, MA, USA), a volume of 20 μL of the diluted sample, standard or PBS (negative control), was added in each well separately with 120 μL of fluorescein (116.66 nM), and then the microplate was preincubated at 37 °C for 10 min. After incubation, 60 μL of 2,2′-azobis-(2-methylpropionamidine) dihydrochloride (AAPH) at 13.02 mg/mL prepared with PBS was added, and then the mixture was incubated at 37 °C for 120 min. Trolox diluted in PBS was used as antioxidant standard (10–80 µM). The fluorescent values were recorded every minute over the incubation period, at 458 nm and 520 nm using a Synergy H1^TM^ microplate reader (BioTek Instruments, Inc., Winooski, VT, USA, EUA) and the experiment was carried out in triplicate.

The area under the curve (AUC) over the incubation period values were calculated by subtracting the AUC of the negative control or blank from all the results. Regression equations between net AUC and antioxidant concentration were calculated. The antioxidant capacity (ORAC value) was expressed in µmoles of Trolox equivalents (TE) per grams of sample and the following equation was used [74]:

(4) ORAC VALUE (µmoles TE/g)=(µmoles TE/L)×DF×(L solvent/g sample)

where *ORAC values* (µmoles TE/L) correspond to the x value obtained from Trolox linear regression (y = mx + b) by replacing y from the AUC sample values, *DF* represents dilution factor, and L solvent/g sample is the volume of prepared mother solution/mass of sample mother solution.

### 3.11. Differential Scanning Calorimetry (DSC)

The thermal characteristics of the samples (melting, crystallization, and decomposition temperatures) were measured on a 204 F1 Phoenix DSC (Netzsch, Selb, Germany). The samples were weighed (4 mg) in a pierced lid aluminium pan and analyzed under a N_2_ flow of 40 mL/min, using the temperature program as follows: First a heating step from 20 to 100 °C to eliminate the sample thermal history [45]; then, a cooling step to −50 °C; followed by a second heating step from 50 to 500 °C. The heating and cooling steps were both performed at a constant rate of 10 °C/min and only the transitions observed during the cooling and the second heating steps were considered.

### 3.12. Statistics

The results are reported as mean values ± standard deviation. Data were first analyzed for normality distribution (i.e., Shapiro–Wilk test). A Levene’s test was applied to verify the homogeneity of the variances. Afterwards, a one-way ANOVA test was applied with Tukey post hoc test to determine differences within groups. For a two-group comparison, Student’s t-test was assayed. The level of significance was set, in general, at 0.05. Analyses of the chromatographic data were performed with the aid of the Jamovi software (v 1.6.3.0, The jamovi project (2020)), retrieved from https://www.jamovi.org; accessed on 7 December 2020).

Analyses of data from cytotoxicity and immune response assays were performed with the aid of Graph Pad Prims 6.0 (Graphpad^®^ software). For PCA and Heatmaps, the web-based tool suite Metaboanalyst (v 5.0) was used (https://www.metaboanalyst.ca/; accessed on 13 January 2021).

## 4. Conclusions

The reported results in this research work showed, for the first time, that biowaste from industrial fermentation activity can be a valuable source of bioactive lipids. Thus, it was possible to recover, through molecular distillation, a fraction enriched in novel triterpenes (acting as coadjuvants) and bioactive lipids as 1-octacosanol and phytosterols. The assayed stability tests highlighted that such mixtures of lipids can undergo oxidation since concentrations of phytosterols decreased during the assayed stability time and 1-octacosanol totally disappeared. Further applications need to devise a procedure to prevent this degradation, namely by avoiding any contact with oxygen. This should be the next step before future studies exploring health applications for these extracts.

However, those changes did not result in in vitro toxicity for macrophages. Moreover, the obtained extracts showed anti-inflammatory capacity as they were able to decrease TNF-α and IL-6 in the presence of LPS, which could be related to the presence of phytosterol and triterpene molecules.

## Figures and Tables

**Table 1:** Lipid class compositions (g/100 g of lipids) of the FDR and feed samples at the different assay times.

FDR	T0			T1			T2			T3		
Compound	Mean		SD	Mean		SD	Mean		SD	Mean		SD
Hydrocarbons	^B^ 43.29	±	0.17	^B^ 44.23	±	1.61	^A^ 52.82	±	0.51	^A^ 54.79	±	0.02
Wax esters	^AB^ 0.22	±	0.05	^A^ 0.23	±	0.01	^B^ 0.17	±	0.01	^C^ 0.14	±	0.02
Triglycerides	^A^ 4.21	±	0.03	^A^ 4.32	±	0.10	^B^ 3.01	±	0.25	^B^ 2.59	±	0.11
Fatty alcohols	^A^ 2.00	±	0.10	^A^ 2.18	±	0.09	^B^ 1.49	±	0.17	^B^ 1.29	±	0.19
Phytosterols	^A^ 0.78	±	0.01	^A^ 0.74	±	0.02	^B^ 0.53	±	0.03	^C^ 0.36	±	0.07
Diglycerides	^A^ 2.62	±	0.12	^A^ 2.51	±	0.02	^B^ 1.67	±	0.13	^B^ 1.61	±	0.02
Free fatty acids	^B^ 0.33	±	0.03	^A^ 0.48	±	0.05	^B^ 0.31	±	0.02	^B^ 0.34	±	0.03
Monoglycerides	^C^0.04	±	0.01	^B^ 0.10	±	0.02	^A^ 0.17	±	0.01	^A^ 0.16	±	0.01
Polymer	^A^ 46.51	±	0.22	^A^ 45.20	±	1.67	^B^ 39.84	±	0.52	^B^ 38.70	±	0.46
**Feed**	**T0**			**T1**			**T2**			**T3**		
**Compound**	**Mean**		**SD**	**Mean**		**SD**	**Mean**		**SD**	**Mean**		**SD**
Hydrocarbons	^B^ 39.54	±	0.92	^B^ 39.71	±	0.91	^A^ 52.60	±	0.62	^A^ 49.46	±	2.34
Wax esters	^A^ 0.11	±	0.01	^A^ 0.11	±	0.02	^B^ 0.07	±	0.01	^C^ 0.06	±	0.01
Triglycerides	^A^ 4.34	±	0.15	^A^ 4.56	±	0.16	^B^ 2.72	±	0.06	^C^ 2.05	±	0.15
Fatty alcohols	^A^ 1.75	±	0.03	^A^ 1.75	±	0.05	^B^ 0.98	±	0.01	^B^ 0.98	±	0.06
Phytosterols	^A^ 0.73	±	0.02	^A^ 0.74	±	0.03	^B^ 0.44	±	0.02	^C^ 0.37	±	0.03
Diglycerides	^A^ 2.08	±	0.05	^A^ 1.95	±	0.20	^B^ 1.22	±	0.05	^C^ 0.90	±	0.22
Free fatty acids	0.30	±	0.04	0.31	±	0.07	0.20	±	0.02	0.22	±	0.01
Monoglycerides	^C^ 0.04	±	0.01	^A^ 0.10	±	0.01	^B^ 0.06	±	0.01	^A^ 0.09	±	0.01
Polymer	^A^ 51.11	±	0.79	^A^ 50.78	±	0.85	^A^ 41.69	±	0.66	^B^ 45.88	±	2.37

Results expressed as mean ± SD (*n* = 3). T0, time 0 (initial); T1, time 1st month; T2, time 2nd month; T3, time 3rd month end of study. Different superscript letters in a row for significant differences along time (*p* < 0.05).

**Table 2:** Profile (g/kg) in terpenes, fatty alcohols, and phytosterols in the FDR and Feed samples at the different assay times.

FDR	T0			T1			T2			T3		
Compound	Mean		SD	Mean		SD	Mean		SD	Mean		SD
β-Farnesene (PR 15)	^A^ 133.48	±	2.81	^B^ 124.35	±	5.56	^C^ 107.19	±	1.11	^D^ 97.15	±	3.1
Farnesol (Z,E) (PR 15:2c2t6)	^A^ 16.26	±	0.38	^B^ 14.95	±	0.38	^B^ 15.10	±	0.92	^C^ 13.02	±	0.48
Farnesol (E,E) (PR 15:2t2t6)	1.36	±	0.04	1.15	±	0.08	1.35	±	0.14	1.07	±	0.11
Sesterterpene (PR 25)	13.5	±	0.28	12.86	±	0.60	11.68	±	1.36	10.53	±	0.76
Triterpene I (PR 30)	^D^ 2.18	±	0.01	^C^ 2.47	±	0.04	^A^ 3.05	±	0.18	^B^ 2.62	±	0.30
Triterpene III (PR 30)	^B^ 2.36	±	0.05	^A^ 3.01	±	0.09	^A^ 3.35	±	0.16	^A^ 3.09	±	0.24
Triterpene IV (PR 30)	^C^ 42.22	±	0.48	^A^ 52.96	±	2.15	^B^ 46.86	±	1.28	^B^ 44.96	±	0.95
Triterpene VI (PR 30)	1.77	±	0.05	2.12	±	0.02	1.30	±	0.25	1.63	±	0.19
Triterpene VIII (PR 30)	^C^ 24.03	±	0.38	^A^ 30.41	±	1.11	^B^ 26.21	±	0.80	^B^ 25.86	±	0.94
Σ TERPENES	^A^ 237.16	±	4.46	^A^ 244.28	±	9.00	^B^ 216.09	±	5.86	^C^ 199.93	±	5.06
1-Octacosanol (FOH 28:0)	1.45	±	0.31	1.13	±	0.08	1.09	±	0.11	0.92	±	0.12
Σ FOH	^A^ 1.45	±	0.31	^B^ 1.13	±	0.08	^B^ 1.09	±	0.11	^B^ 0.92	±	0.12
Ergosterol (ST 28:3;O)	^B^ 3.19	±	0.14	^A^ 3.72	±	0.09	^B^ 3.07	±	0.26	^C^ 1.70	±	0.22
Campesterol (ST 28:1;O)	0.72	±	0.05	0.86	±	0.01	0.94	±	0.07	0.77	±	0.09
Stigmasterol (ST 29:2;O)	0.92	±	0.04	1.03	±	0.02	1.07	±	0.12	0.98	±	0.11
β-Sitosterol (ST 29:1;O)	1.29	±	0.05	1.51	±	0.05	1.60	±	0.15	1.47	±	0.15
Σ ST	^A^ 6.11	±	0.28	^A^ 7.11	±	0.14	^A^ 6.68	±	0.59	^B^ 4.92	±	0.57
**Feed**	**T0**			**T1**			**T2**			**T3**		
**Compound**	**Mean**		**SD**	**Mean**		**SD**	**Mean**		**SD**	**Mean**		**SD**
β-Farnesene (PR 15)	^B^ 116.09	±	1.52	^A^ 119.95	±	2.5	^C^ 101.27	±	0.7	^D^ 66.30	±	1.46
Farnesol (Z,E) (PR 15:2c2t6)	^A^ 15.67	±	0.17	^A^ 16.47	±	0.21	^A^ 16.74	±	0.15	^B^ 14.74	±	0.63
Farnesol (E,E) (PR 15:2t2t6)	1.27	±	0.03	1.33	±	0.02	1.49	±	0.02	1.24	±	0.03
Sesterterpene (PR 25)	14.83	±	0.14	15.14	±	0.37	13.56	±	0.03	13.04	±	0.80
Triterpene I (PR 30)	^C^ 2.08	±	0.14	^C^ 2.67	±	0.12	^A^ 3.08	±	0.12	^B^ 2.98	±	0.20
Triterpene III (PR 30)	^B^ 2.61	±	0.12	^A^ 2.84	±	0.34	^B^ 3.27	±	0.09	^B^ 3.16	±	0.11
Triterpene IV (PR 30)	^B^ 45.69	±	0.46	^A^ 50.11	±	0.45	^C^ 44.94	±	0.63	^C^ 43.95	±	1.45
Triterpene VI (PR 30)	^A^ 2.02	±	0.06	^A^ 2.44	±	0.08	^C^ 1.57	±	0.06	^B^ 1.82	±	0.16
Triterpene VIII (PR 30)	^B^ 25.99	±	0.26	^A^ 28.64	±	0.36	^C^ 25.05	±	0.33	^C^ 24.84	±	0.86
Σ TERPENES	^A^ 226.26	±	0.13	^A^ 239.59	±	1.39	^B^ 210.97	±	1.75	^C^ 172.08	±	5.46
1-Octacosanol (FOH 28:0)	0.25	±	0.11	0.23	±	0.01	0.24	±	0.02	0.21	±	0.02
Σ FOH	0.25	±	0.11	0.23	±	0.01	0.24	±	0.02	0.21	±	0.02
Ergosterol (ST 28:3;O)	^B^ 3.54	±	0.12	^A^ 3.70	±	0.17	^B^ 3.00	±	0.14	^C^ 1.79	±	0.16
Campesterol (ST 28:1;O)	0.71	±	0.01	0.78	±	0.05	0.88	±	0.03	0.83	±	0.06
Stigmasterol (ST 29:2;O)	0.90	±	0.03	1.13	±	0.02	1.22	±	0.06	1.23	±	0.14
β-Sitosterol (ST 29:1;O)	1.36	±	0.08	1.69	±	0.1	1.79	±	0.08	1.76	±	0.14
Σ ST	^A^ 6.52	±	0.24	^A^ 7.31	±	0.29	^A^ 6.89	±	0.17	^B^ 5.62	±	0.49

Results expressed as mean ± SD (*n* = 3). T0, time 0 (initial); T1, time 1st month; T2, time 2nd month; T3, time 3rd month end of study. FOH, fatty alcohols and ST, sterols. Different superscript letters in a row for significant differences along time (*p* < 0.05).

**Table 3:** Profile (g/kg) in terpenes, fatty alcohols, and phytosterols in distilled samples (C6 and C7) at the different assay times.

C6	T0			T1			T2			T3		
Compound	Mean		SD	Mean		SD	Mean		SD	Mean		SD
Sesterterpene (PR 25)	^A^ 3.40	±	0.20	^A^ 3.38	±	0.24	^B^ 2.28	±	0.05	^B^ 2.15	±	0.21
Triterpene I (PR 30)	^B^ 22.86	±	2.93	^A^ 29.15	±	0.60	^B^ 26.23	±	0.76	^B^ 24.35	±	0.26
Triterpene II (PR 30)	^C^ 3.32	±	0.27	^B^ 4.57	±	0.28	^A^ 6.38	±	0.54	^D^ 3.90	±	0.22
Triterpene III (PR 30)	^B^ 33.36	±	3.74	^A^ 41.27	±	0.36	^B^ 36.79	±	1.29	^B^ 34.74	±	0.08
Triterpene IV (PR 30)	^B^ 438.12	±	19.4	^A^ 504.66	±	0.44	^C^ 404.35	±	8.84	^C^ 411.04	±	6.10
Triterpene V (PR 30)	^C^ 5.82	±	0.25	^C^ 5.74	±	0.13	^A^ 14.26	±	1.23	^B^ 7.61	±	1.25
Triterpene VII (PR 30)	^A^ 7.42	±	0.51	^A^ 7.90	±	0.51	^A^ 8.81	±	1.53	^B^ 6.50	±	0.03
Triterpene VIII (PR 30)	^B^ 242.75	±	13.2	^A^ 282.45	±	3.14	^D^ 184.12	±	2.83	^C^ 220.45	±	8.02
Σ TERPENES	^B^ 757.05	±	40.5	^A^ 879.12	±	3.7	^C^ 680.93	±	17	^C^ 710.74	±	13.1
1-Octacosanol (FOH 28:0)	^A^ 1.61	±	0.18	^B^ 1.49	±	0.06	^C^ <LOD		na	^C^ <LOD		na
Σ FOH	^A^ 1.61	±	0.18	^B^ 1.49	±	0.06	^C^ <LOD		na	^C^ <LOD		na
Ergosterol (ST 28:3;O)	^B^ 5.91	±	0.98	^A^ 9.93	±	0.28	^C^ 2.20	±	0.35	^C^ 2.51	±	0.21
Campesterol (ST 28:1;O)	^C^ 1.64	±	0.01	^A^ 2.70	±	0.1	^B^ 2.01	±	0.33	^D^ 1.12	±	0.06
Stigmasterol (ST 29:2;O)	^C^ 1.67	±	0.06	^A^ 2.81	±	0.04	^B^ 2.30	±	0.01	^D^ 1.37	±	0.14
β-Sitosterol (ST 29:1;O)	^B^ 2.18	±	0.25	^A^ 3.08	±	0.21	^C^ 1.67	±	0.13	^C^ 1.61	±	0.04
Σ ST	^B^ 11.40	±	1.27	^A^ 18.53	±	0.53	^C^ 8.18	±	0.82	^D^ 6.61	±	0.37
**C7**	**T0**			**T1**			**T2**			**T3**		
**Compound**	**Mean**		**SD**	**Mean**		**SD**	**Mean**		**SD**	**Mean**		**SD**
Triterpene I (PR 30)	^C^ 9.80	±	1.19	^A^ 11.48	±	0.2	^C^ 9.67	±	0.54	^B^ 10.66	±	0.52
Triterpene II (PR 30)	^C^ 6.65	±	0.91	^A^ 8.15	±	0.27	^C^ 6.82	±	0.2	^B^ 6.95	±	0.26
Triterpene III (PR 30)	^D^ 20.30	±	2.13	^A^ 23.66	±	0.32	^C^ 21.08	±	0.34	^B^ 21.45	±	0.87
Triterpene IV (PR 30)	^A^ 352.42	±	23.4	^A^ 383.56	±	1.47	^C^ 309.84	±	3.85	^B^ 328.42	±	5.28
Triterpene V (PR 30)	9.41	±	0.90	9.61	±	0.25	9.95	±	0.91	9.76	±	0.34
Triterpene VII (PR 30)	5.26	±	0.69	5.57	±	0.35	4.83	±	0.62	3.88	±	0.20
Triterpene VIII (PR 30)	^B^ 230.35	±	13.1	^A^ 252.10	±	1.64	^D^ 195.89	±	6.00	^C^ 201.61	±	5.36
Σ TERPENES	^B^ 634.20	±	42.3	^A^ 694.14	±	3.74	^D^ 558.09	±	9.2	^C^ 582.73	±	9.08
1-Octacosanol (FOH 28:0)	^A^ 3.88	±	0.36	^B^ 3.42	±	0.03	^C^ <LOD		na	^C^ <LOD		na
Σ FOH	^A^ 3.88	±	0.36	^B^ 3.42	±	0.03	^C^ <LOD		na	^C^ <LOD		na
Ergosterol (ST 28:3;O)	^B^ 15.93	±	1.82	^A^ 18.95	±	0.28	^C^ 11.21	±	0.82	^D^ 5.88	±	0.25
Campesterol (ST 28:1;O)	^B^ 4.20	±	0.39	^A^ 5.11	±	0.27	^C^ 3.91	±	0.27	^D^ 2.83	±	0.07
Stigmasterol (ST 29:2;O)	^B^ 4.63	±	0.45	^A^ 5.68	±	0.04	^C^ 3.85	±	0.33	^D^ 3.12	±	0.15
β-Sitosterol (ST 29:1;O)	^B^ 6.08	±	0.79	^A^ 7.04	±	0.29	^C^ 4.50	±	0.05	^C^ 3.61	±	0.16
Σ ST	^B^ 30.84	±	3.45	^A^ 36.77	±	0.64	^C^ 23.47	±	1.33	^D^ 15.44	±	0.38

Results expressed as mean ± SD (*n* = 3). T0, time 0 (initial); T1, time 1st month; T2, time 2nd month; T3, time 3rd month end of study. FOH, fatty alcohols and ST, sterols. Different superscript letters in a row for significant differences during the assayed time (*p* < 0.05).

**Table 4:** Lipid class compositions (g/100 g of lipids) of distilled samples (C6 and C7) at the different assay times.

C6	T0			T1			T2			T3		
Compound	Mean		SD	Mean		SD	Mean		SD	Mean		SD
Hydrocarbons	^C^ 90.93	±	0.64	^B^ 91.42	±	0.70	^A^ 92.68	±	0.23	^A^ 93.50	±	0.16
Wax esters	^B^ 0.21	±	0.01	^A^ 0.42	±	0.02	^B^ 0.24	±	0.12	^B^ 0.37	±	0.01
Triglycerides	^A^ 0.98	±	0.10	^A^ 0.86	±	0.09	^B^ 0.73	±	0.03	^C^ 0.52	±	0.05
Fatty alcohols	^A^ 3.11	±	0.24	^A^ 3.03	±	0.28	^B^ 2.60	±	0.12	^B^ 2.47	±	0.14
Phytosterols	^A^ 1.31	±	0.10	^A^ 1.25	±	0.09	^B^ 1.13	±	0.01	^B^ 1.02	±	0.03
Diglycerides	^A^ 3.01	±	0.08	^B^ 2.42	±	0.24	^B^ 2.41	±	0.05	^C^ 2.01	±	0.02
Free fatty acids	^B^ 0.46	±	0.11	^A^ 0.60	±	0.09	^C^ 0.21	±	0.01	^D^ 0.11	±	0.01
**C7**	**T0**			**T1**			**T2**			**T3**		
**Compound**	**Mean**		**SD**	**Mean**		**SD**	**Mean**		**SD**	**Mean**		**SD**
Hydrocarbons	^C^ 85.81	±	0.31	^A^ 87.52	±	0.59	^B^ 87.79	±	0.25	^C^ 85.77	±	0.33
Wax esters	^B^ 0.41	±	0.01	^A^ 0.52	±	0.04	^C^ 0.29	±	0.01	^B^ 0.46	±	0.01
Triglycerides	^A^ 1.29	±	0.15	^A^ 0.98	±	0.05	^B^ 0.80	±	0.03	^B^ 0.78	±	0.01
Fatty alcohols	^A^ 3.59	±	0.59	^A^ 3.39	±	0.03	^B^ 2.47	±	0.10	^B^ 2.34	±	0.07
Phytosterols	^A^ 3.40	±	0.08	^B^ 3.30	±	0.06	^B^ 3.06	±	0.10	^C^ 2.89	±	0.07
Diglycerides	4.68	±	1.09	4.35	±	0.32	5.42	±	0.08	5.76	±	0.21
Free fatty acids	^B^ 0.82	±	0.04	^A^ 1.11	±	0.13	^B^ 0.46	±	0.01	^C^ 0.55	±	0.02

Results expressed as mean ± SD (*n* = 3). T0, time 0 (initial); T1, time 1st month; T2, time 2nd month; T3, time 3rd month end of study. Different superscript letters in a row for significant differences during the assayed time (*p* < 0.05).

**Table 5:** Thermal characteristics (temperatures and enthalpies) of the study samples throughout the stability test.

	Temperature (°C) (|ΔH| (J/g))		
FDR	Crystallization	Melting	Decomposition
T0	40.9 (4.7)	44.1(6.4)	400.5 (553.9)
T1	na	na	401.5 (317.2)
T2	na	na	408.1 (536.5)
T3	na	na	403.9 (324.1)
**Temperature (°C) (|ΔH| (J/g))**			
**Feed**	**Crystallization**	**Melting**	**Decomposition**
T0	29.6 (1.6)	39.4 (4.3)	400.7 (373.5)
T1	na	na	399.2 (348.4)
T2	na	na	399.4 (409.7)
T3	na	na	399.2 (295.7)
**Temperature (°C) (|ΔH| (J/g))**			
C6	**Crystallization**	**Melting**	**Decomposition**
T0	28.6 (2.6)	41.5 (2.7)	365.3 (215.5)
T1	28.3 (2.2)	39.3 (2.2)	383.9 (180.8)
T2	27.1 (2.2)	37.9 (2.3)	374.5 (172.9)
T3	26.7 (1.8)	35.4 (1.7)	373.5 (137.2)
**Temperature (°C) (|ΔH| (J/g))**			
C7	**Crystallization**	**Melting**	**Decomposition**
T0	37.2 (5.3)	46.4 (5.4)	370.9 (297.3)
T1	39.3 (4.4)	47.4 (4.7)	377.4 (117.9)
T2	38.2 (3.7)	47.6 (5.4)	371.6 (158.2)
T3	35.8 (4.1)	44.2 (3.4)	371.8 (166.3)

na, not available.

## Data Availability

Data is contained within the article or Supplementary Materials.

## Supplementary Materials

The following are available online at https://www.mdpi.com/article/10.3390/ph14060583/s1, Supplementary Methods and Materials (Polymer isolation from sample, Shotgun QTOF, LC-MS analyses), Figure S1. Chromatographic overlay of the triterpene eluting region of the assayed samples, Figure S2. Mass spectra (GC-MS) of triterpene I in the assayed samples, Figure S3. Mass spectra (GC-MS) of triterpene III in the assayed samples, Figure S4. Mass spectra (GC-MS) of triterpene IV in the assayed samples, Figure S5. Mass spectra (GC-MS) of triterpene VIII in the assayed samples, Figure S6. Mass spectra (GC-MS) of squalene (Standard from Sigma), Figure S7. Direct infusion on QTOF of FDR sample treated with methanol showed a gaussian distribution of ions, Figure S8. Mass spectra (LC-QTOF) at start (A), middle (B) and end (C) of eluting peak of methanol-treated FDR sample, Figure S9. PCA of data obtained from GC-MS (A) and HPLC-ELSD (B), Figure S10. Heatmap of GC-MS data from the assayed samples, Figure S11. Heatmap of HPLC-ELSD data from the assayed samples, Table S1. Mobile phase gradient (%), Table S2. FTIR frequencies interpretation table, Table S3. Quantification of IL-1β, IL-6, IL-8 and TNF-α levels in macrophages without and with inflammatory stimulus (LPS), Supplemetary References.

## Abbreviations

Phospholipids (PLS), triglycerides (TG), diglycerides (DG), free fatty acids (FFA), fatty alcohols (FOH), 1-octacosanol (FOH 28:0), dichloromethane (DCM), ethanol (EtOH), acetone (AcO) or ethyl acetate (EtAc), triethylamine (TEA), N, O-Bis(trimethylsilyl) trifluoroacetamide with 1% trimethylchlorosilane (BSTFA), trimethylsilyl derivatives (TMS), fatty aldehydes (FAL), sterols (ST), phosphatidylethanolamine (PE), phosphatidylcholine (PC), phosphatidylinositol (PI), tritriacontane (C33:0), campesterol (ST C28:1;O), stigmasterol (ST 29:2;O), β-sitosterol (ST 29:1;O), and stigmast-4-en-3-one (ST29:2;O2).
