# Supplementary material for: Phytosterols and Novel Triterpenes Recovered from Industrial Fermentation Coproducts Exert In Vitro Anti-Inflammatory Activity in Macrophages
Source: Pharmaceuticals (Basel). 2021 Jun 18;14(6):583. doi: 10.3390/ph14060583 (PMC8235040; doi:10.3390/ph14060583)
Supplement: Supplementary file 1 [file pharmaceuticals-14-00583-s001.zip › pharmaceuticals-1210699-supplementary.pdf]

Supplementary Methods and Materials

Polymer isolation from sample

Using a beaker, add methanol in a proportion of 1:4 (w:v) to the sample. Afterwards mixture is blended for 5 min and let stand at room temperature for 2h. Then, it is placed at –30 °C overnight. Next, the sample is centrifuged at 1250 g for 10 min at room temperature. The Solvent is collected and eliminated in a rotatory evaporator.

Shotgun QTOF

Sample was prepared in isopropanol at 1 mg/mL with 0.1% formic acid. Then, 1 µL was directly injected in Impact II QTOF (Bruker, Karlsruhe, Germany). Ionization voltage was +4.5 kV in positive, end plate offset 0.5 kV mode; nebulizing gas pressure was 1.8 bar (8 L/min) and drying temperature 220 ºC. Spectra were acquired within the range of m/z 150–2000.

LC-MS analyses

Analyses conditions were as reported by Sarafian et al. [1]. Thus, chromatographic analyses were performed using an ELUTE LC series system (Bruker, Karlsruhe, Germany) coupled to an Impact II QTOF (Bruker, Karlsruhe, Germany). Separation of compounds was conducted using a C18 Intensity Solo column (100 mm x 2.1 mm, 2 µm) at 55 °C and 0.400 mL/min. A binary mobile system was used for the elutions of compounds i) phase A consists of ACN/H_2O (60:40, v:v) mixed with 10 mM ammonium formate and 0.1% formic acid and ii) mobile phase B IPA/ACN (90:10, v:v) mixed with 10 mM ammonium formate and 0.1% formic acid. Gradient was as described by Sarafian et al [1]. The injection volume was 3 µL. FDR samples treated with methanol were dissolved at a concentration of 0.3 mg/mL in phase B and analyzed in positive mode: Ionization voltage was +4.5 kV, end plate offset 0.5 kV; nebulizing gas pressure was 1.8 bar (8 L/min) and drying temperature 220 ºC; spectra were acquired within the range of m/z 150–2000.

Supplementary results

Figure S1. Chromatographic overlay of the triterpene eluting region of the assayed samples.

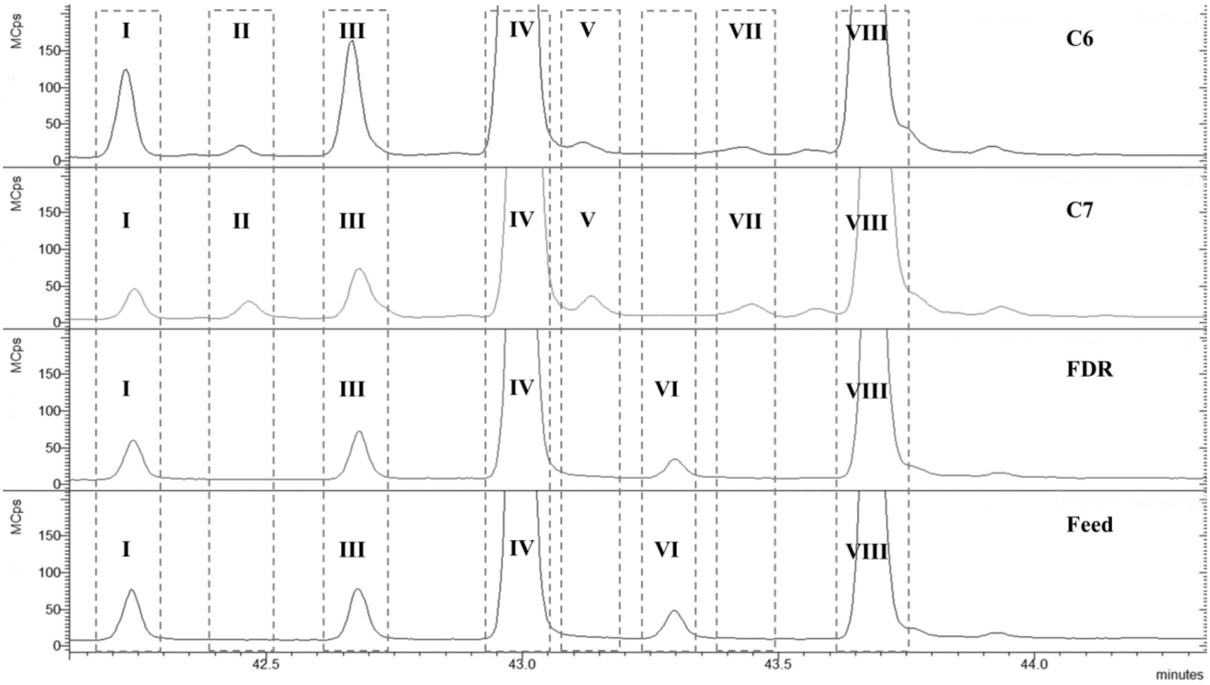

Figure S2. Mass spectra (GC-MS) of triterpene I in the assayed samples.

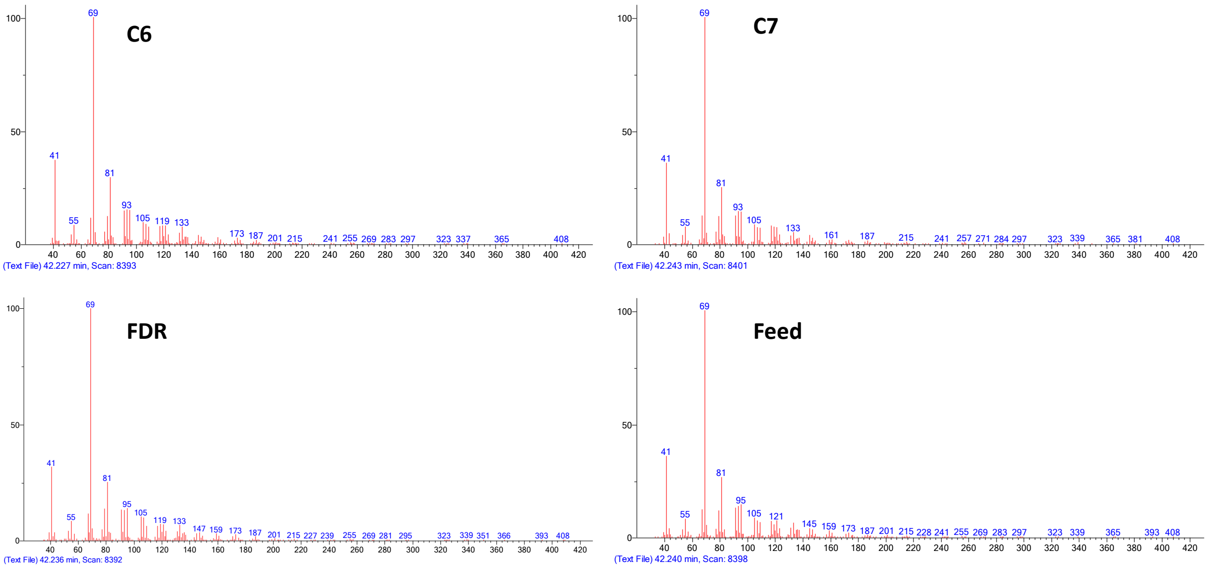

Figure S3. Mass spectra (GC-MS) of triterpene III in the assayed samples.

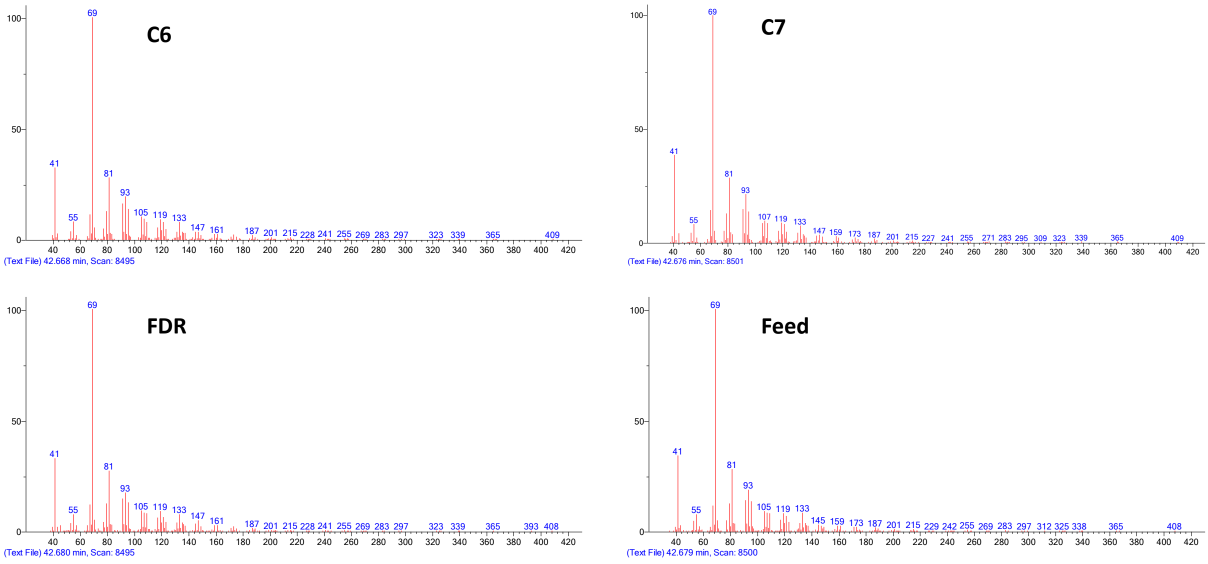

Figure S4. Mass spectra (GC-MS) of triterpene IV in the assayed samples.

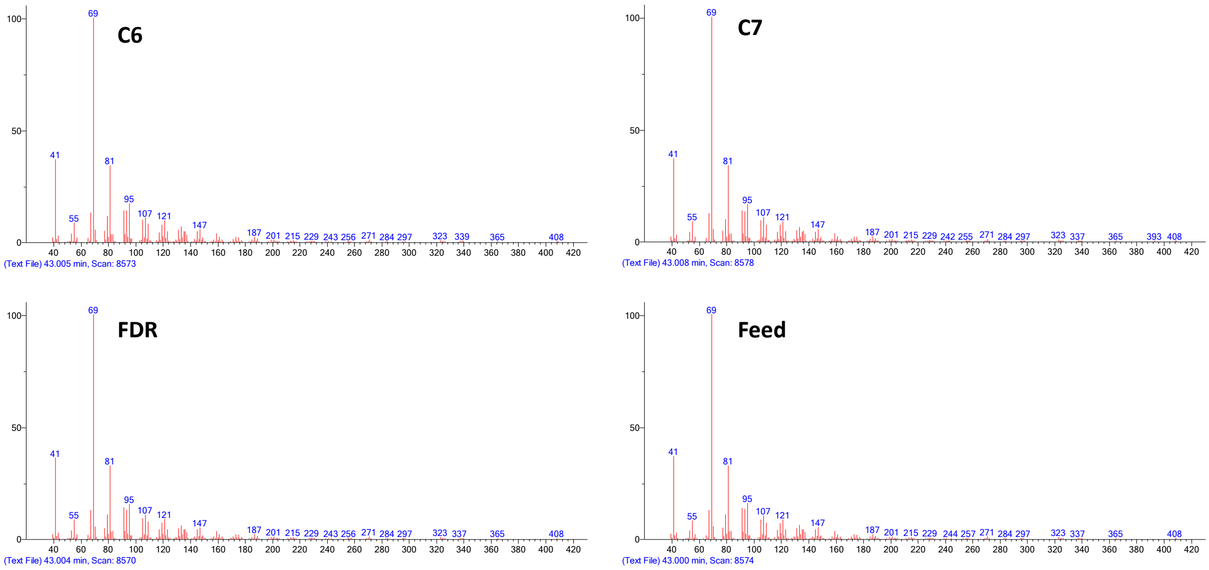

Figure S5. Mass spectra (GC-MS) of triterpene VIII in the assayed samples.

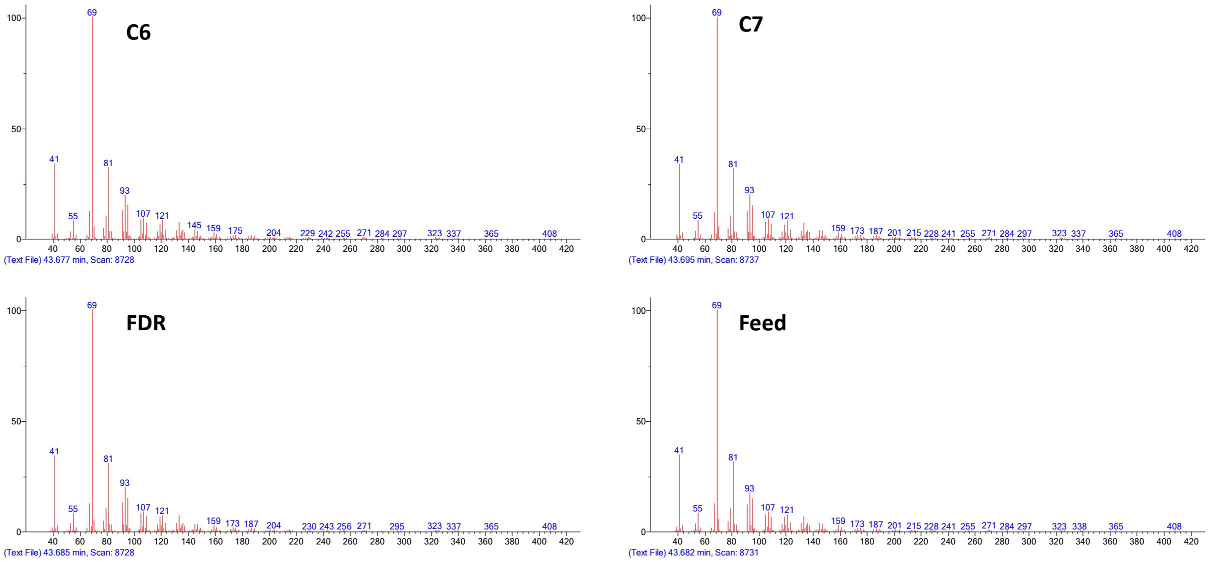

Figure S6. Mass spectra (GC-MS) of squalene (Standard from Sigma).

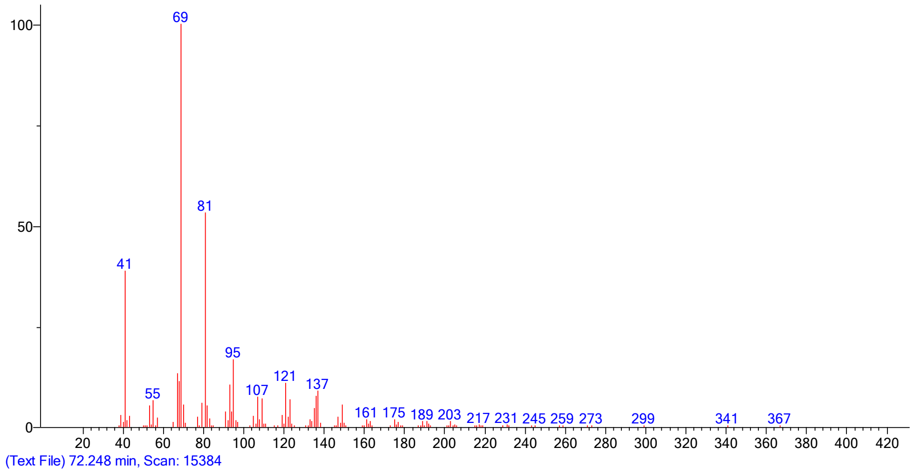

Figure S7. Direct infusion on QTOF of FDR sample treated with methanol showed a gaussian distribution of ions.

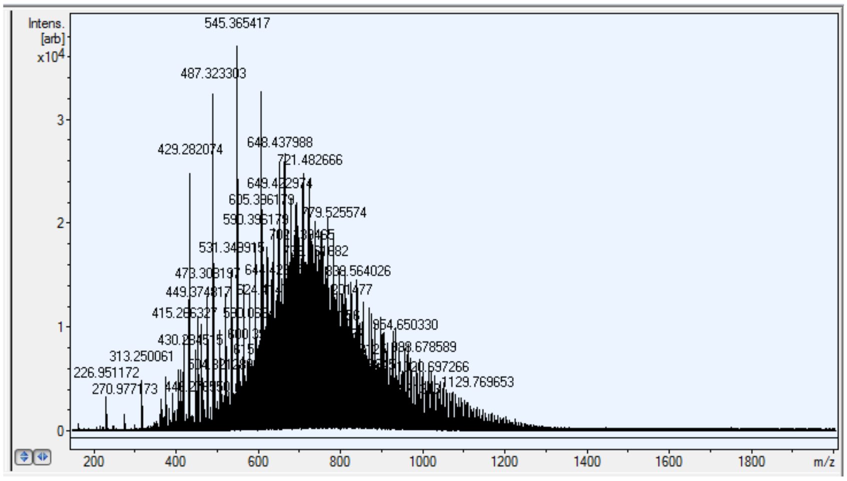

Figure S8. Mass spectra (LC-QTOF) at start (A), middle (B) and end (C) of eluting peak of methanol-treated FDR sample.

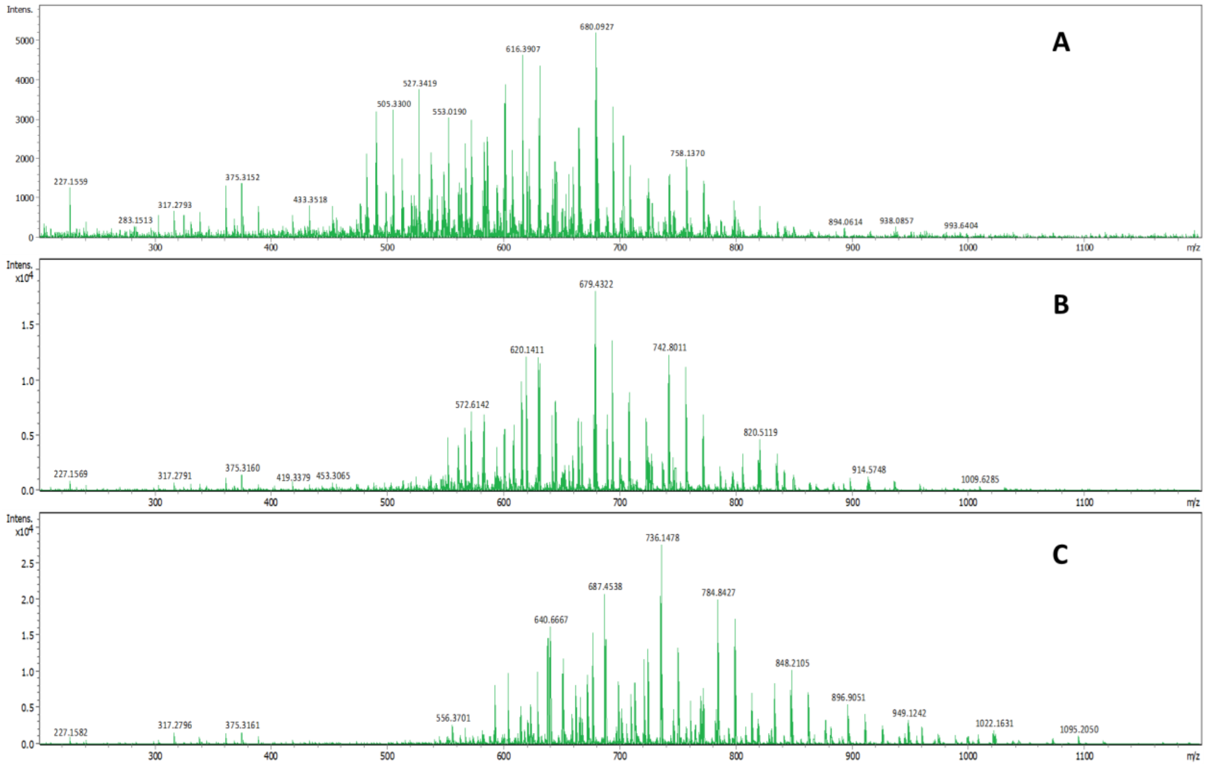

Figure S9. PCA of data obtained from GC-MS (A) and HPLC-ELSD (B).

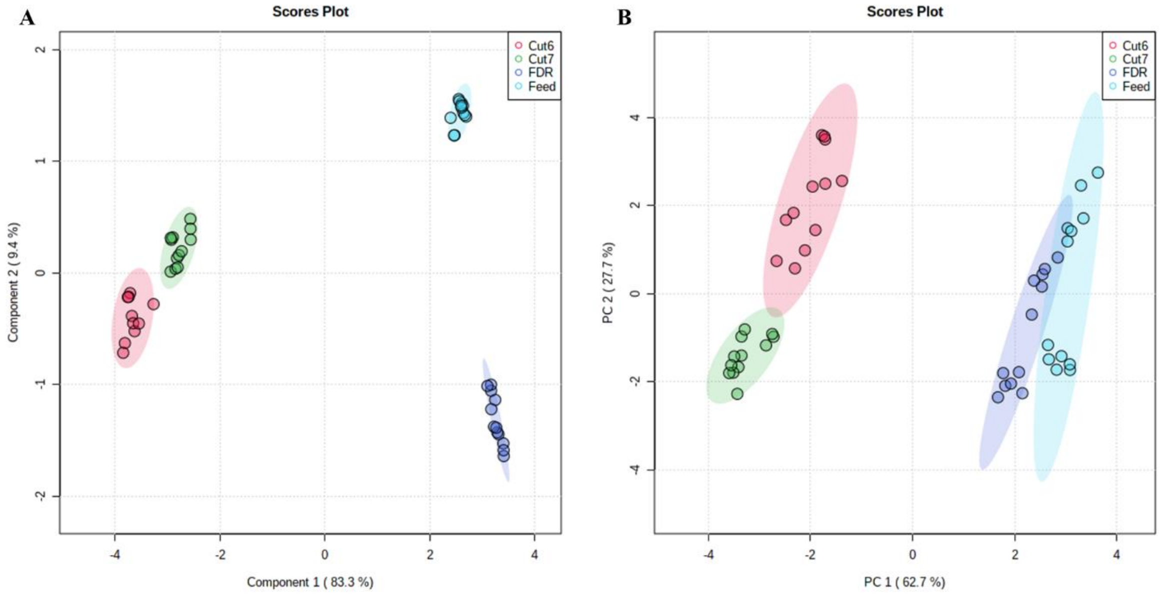

Figure S10. Heatmap of GC-MS data from the assayed samples.

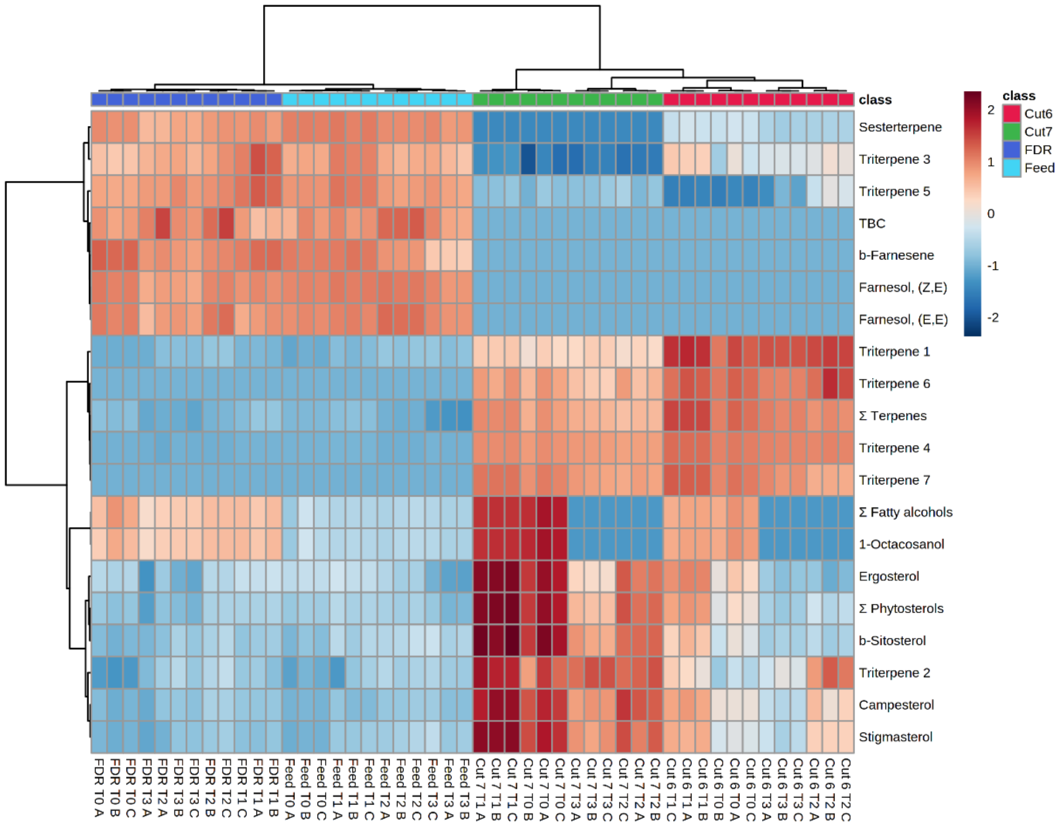

Figure S11. Heatmap of HPLC-ELSD data from the assayed samples.

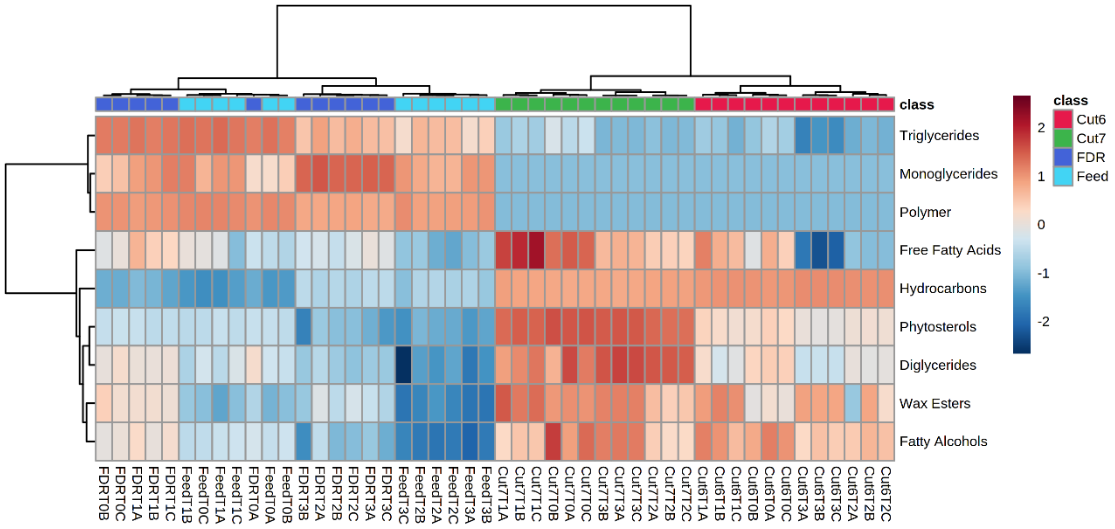

Table S1.Mobile phase gradient (%).

**Time (min.)**	**Mobile phase (percent)**				**Flow-rate (mL/min.)**
	**A**	**B**	**C**	**D**	
0.0	100	0	0	0	0.275
1.5	100	0	0	0	0.275
1.6	97	3	0	0	0.275
9.0	94	6	0	0	0.275
11.0	70	30	0	0	0.275
14.0	45	55	0	0	0.275
15.0	45	55	0	0	0.275
16.0	40	55	5	0	0.275
20.0	35	55	10	0	0.275
20.1	33	50	17	0	0.275
25.0	38	45	17	0	0.275
25.1	48	35	17	0	0.275
30.0	53	30	17	0	0.275
40.0	40	0	60	0	0.275
40.1	0	100	0	0	0.275
42.0	0	100	0	0	0.275
42.1	50	0	0	50	0.275
45.0	50	0	0	50	0.275
47.0	100	0	0	0	0.275
55.0	100	0	0	0	0.275

Table S2. FTIR frequencies interpretation table.

**Wavenumber (cm^{-1})**	**Origin**	**Assignment**
3486-3390	-OH stretching	Alcohols
2969-2849	C-H stretching (-CH_3, -CH_2 and -CH)	Aliphatic chains
1741-1738	-C=O stretching	Esters, Carboxylic Acids, Ketones, Aldehydes
1714-1712	-OH bending	Alcohols
1671<br>1641	C=C stretching	Unsaturated aliphatic chains
1595	$RONH_2$	Amines
1451-1374	C-H bending	Aliphatic chains
1347	CH_3 deformation	Aliphatic chains
1297	(unsat.) -CH deformation	Unsaturated aliphatic chains
~1251	CH_3 symmetric deformation	Aliphatic chains
1108-1102<br>1014	CH_3-CO Rocking	Ketones
984	CH deformation	Aliphatic chains
904-892	CH deformation (out of plane)	Aliphatic chains
~888	CH_2 out of plane deformation	Unsaturated aliphatic chains
836-833	CH_2 rocking vibration	Aliphatic chains
744	CH_2 twisting	Unsaturated aliphatic chains
719	Rotational deformation of CH_2 in chain	High aliphatic chains

Table S3.Quantification of IL-1β, IL-6, IL-8 and TNF-α levels in macrophages without and with inflammatory stimulus (LPS).

	No stimulus	LPS stimulus
**IL-1β**		
Untreated cells	0.38±0.08	6.32±0.19
Ibuprofen	-	8.47±0.36***
C6 T0	0.25±0.05	7.15±0.23
C6 T3	0.32±0.06	7.40±0.56
C7 T0	0.18±0.08	6.98±0.15
C7 T3	0.18±0.05	7.10±0.11
**IL-6**		
Untreated cells	n.d.	76.8±7.4
Ibuprofen	-	24.9±5.0****
C6 T0	n.d.	5.00±0.26****
C6 T3	n.d.	4.40±0.40****
C7 T0	n.d.	5.05±0.40****
C7 T3	n.d.	4.58±0.67****
**IL-8**		
Untreated cells	17.4±0.9	219±32
Ibuprofen	-	195±31
C6 T0	24.5±0.1****	255±44
C6 T3	28.8±0.6****	237±44
C7 T0	22.3±0.7***	221±38
C7 T3	18.9±0.4	236±48
**TNF-α**		
Untreated cells	0.42±0.05	96.7±6.9
Ibuprofen	-	76.0±4.8
C6 T0	0.40±0.01	60.2±8.1**
C6 T3	0.45±0.06	57.7±6.4**
C7 T0	0.28±0.02	58.9±8.7**
C7 T3	0.20±0.04**	56.0±7.4**

Values are expressed as pg of cytokine normalized to the total protein content. Ibuprofen at 1 mM was used as an anti-inflammatory control. One-way ANOVA was used for statistical analyses.

Supplemetary References

1. Sarafian, M.H.; Gaudin, M.; Lewis, M.R.; Martin, F.P.; Holmes, E.; Nicholson, J.K.; Dumas, M.E. Objective set of criteria for optimization of sample preparation procedures for ultra-high throughput untargeted blood plasma lipid profiling by ultra performance liquid chromatography-mass spectrometry. *Anal. Chem.* **2014**, *86*, 5766–5774, doi:10.1021/ac500317c.
